# Chemical Proteomics Reveals Off-Targets of the Anandamide Reuptake Inhibitor WOBE437

**DOI:** 10.1021/acschembio.2c00122

**Published:** 2022-04-28

**Authors:** Berend Gagestein, Anna F. Stevens, Domenico Fazio, Bogdan I. Florea, Tom van der Wel, Alexander T. Bakker, Filomena Fezza, Hans den Dulk, Herman S. Overkleeft, Mauro Maccarrone, Mario van der Stelt

**Affiliations:** † Department of Molecular Physiology, Leiden Institute of Chemistry, Leiden University, Einsteinweg 55, Leiden 2333 CC, The Netherlands; ‡ European Center for Brain Research/IRCCS Santa Lucia Foundation, Via del Fosso di Fiorano 64, Rome 00143, Italy; § Department of Experimental Medicine, Tor Vergata University of Rome, Via Montpellier 1, Rome 00121, Italy; ∥ Bio-Organic Synthesis, Leiden Institute of Chemistry, Leiden University, Einsteinweg 55, Leiden 2333 CC, The Netherlands; ⊥ Department of Biotechnological and Applied Clinical Sciences, University of L’Aquila, Via Vetoio snc, 67100 L’Aquila, Italy

## Abstract

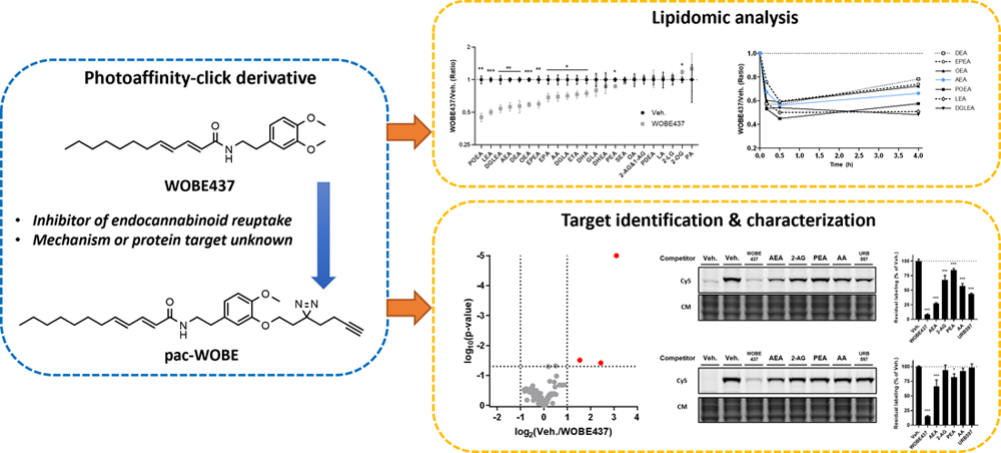

Anandamide or *N*-arachidonoylethanolamine (AEA) is a signaling lipid that modulates neurotransmitter release via activation of the type 1 cannabinoid receptor (CB_1_R) in the brain. Termination of anandamide signaling is thought to be mediated *via* a facilitated cellular reuptake process that utilizes a purported transporter protein. Recently, WOBE437 has been reported as a novel, natural product-based inhibitor of AEA reuptake that is active in cellular and *in vivo* models. To profile its target interaction landscape, we synthesized pac-WOBE, a photoactivatable probe derivative of WOBE437, and performed chemical proteomics in mouse neuroblastoma Neuro-2a cells. Surprisingly WOBE437, unlike the widely used selective inhibitor of AEA uptake OMDM-1, was found to increase AEA uptake in Neuro-2a cells. In line with this, WOBE437 reduced the cellular levels of AEA and related *N*-acylethanolamines (NAEs). Using pac-WOBE, we identified saccharopine dehydrogenase-like oxidoreductase (SCCPDH), vesicle amine transport 1 (VAT1), and ferrochelatase (FECH) as WOBE437-interacting proteins in Neuro-2a cells. Further genetic studies indicated that SCCPDH and VAT1 were not responsible for the WOBE437-induced reduction in NAE levels. Regardless of the precise mechanism of action of WOB437 in AEA transport, we have identified SSCPHD, VAT1, and FECH as unprecedented off-targets of this molecule which should be taken into account when interpreting its cellular and *in vivo* effects.

## Introduction

Anandamide (AEA) is a lipid signaling molecule that belongs to the endocannabinoid system (ECS). It modulates neurotransmitter release *via* activation of the type 1 cannabinoid receptor (CB_1_R).^1^ AEA is produced by hydrolysis of phospholipids, mainly by *N*-acylphosphatidylethanolamine-specific phospholipase D (NAPE-PLD), after which it is released to activate CB_1_R. AEA-induced CB_1_R signaling is terminated by a two-step process, that is, cellular uptake followed by hydrolysis of the amide bond by fatty acid amide hydrolase (FAAH). The ECS is responsible for the regulation of a large number of pathophysiological processes, including energy balance, pain, inflammation, and neurotransmission.^2^ Consequently, modulation of ECS signaling may have therapeutic benefits for a number of diseases, including neurodegenerative,^3,4^ inflammatory,^5^ and cardiovascular diseases,^6−8^ pain,^9,10^ psychiatric disorders,^2,11^ obesity,^12^ and others.^13^ Activation of CB_1_R signaling has been achieved by direct and indirect methods, that is, through the application of CB_1_R agonists and through altering endocannabinoid metabolism, respectively.^13^ The latter strategy may lead to fewer side effects that are usually associated with direct CB_1_R activation.^14^

Elevation of AEA levels can be achieved by inhibiting its reuptake across the plasma membrane or by inhibiting its hydrolysis through FAAH. While inhibition of FAAH is well-characterized and selective inhibitors are currently tested in phase 2 clinical trials, the mechanism of AEA reuptake remains unclear. Small lipophilic molecules may diffuse freely through the lipid bilayer, but AEA reuptake can be saturated.^15^ This indicates that a protein facilitator for transport across the membrane may exist. Numerous candidates for a purported endocannabinoid membrane transporter and their inhibitors (AM404,^16^ VDM11,^17^ UCM707,^18^ and OMDM-1/2^19^) have been reported, but the existence of such a transporter remains as yet a subject of intense scientific debate.^20−25^ One of the difficulties to solve the issue is the technical challenge of reliably measuring AEA uptake in short timeframes.^24^ Moreover, FAAH inhibition results in accumulation of intracellular AEA, which disrupts the concentration gradient across the cellular membrane that normally drives AEA uptake from the extracellular milieu.^25^ Unsurprisingly, many AEA uptake inhibitors have been revealed to act through inhibition of FAAH.^25,26^ Another confounding factor is inhibition of intracellular trafficking of AEA, which can also reduce AEA reuptake. For example, inhibition of FABP5, which is an intracellular binding protein that transports AEA to FAAH at the endoplasmic reticulum,^27^ blocks AEA uptake.^28^ Other intracellular AEA binding proteins are Hsp70,^29^ albumin, and potentially FLAT, a catalytically inactive version of FAAH.^30^

**Figure 1:**
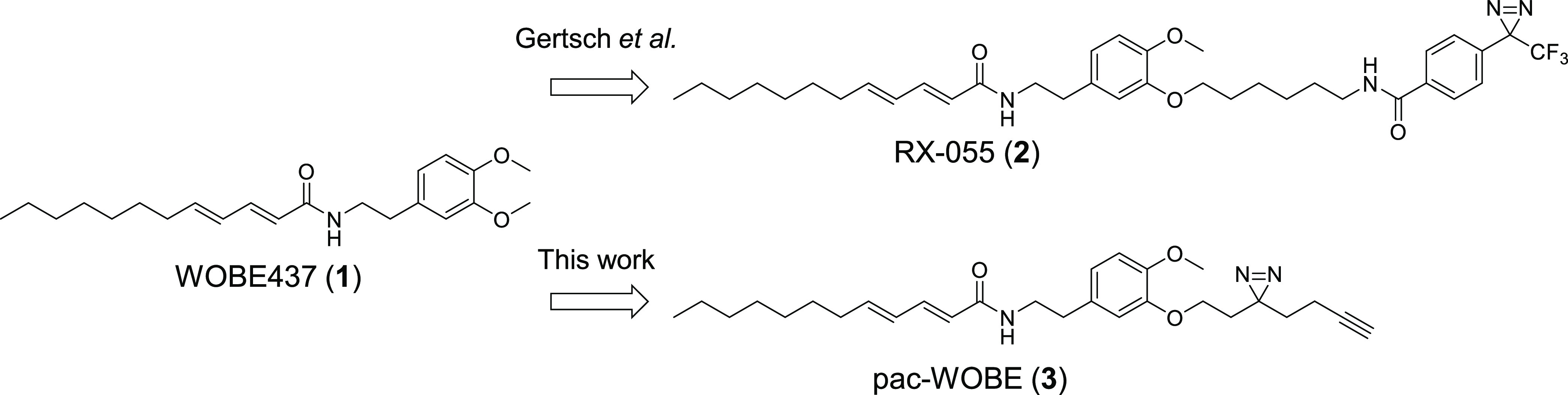
Structures of WOBE437 and its probe derivatives.

Recently, WOBE437 (**1**, Figure 1) has been reported by Dr. Gertsch and co-workers as a novel, natural product-based AEA uptake inhibitor, which is selective over FAAH, FABP5, Hsp70, and FLAT.^31^ WOBE437 reduced AEA uptake in mouse neuroblastoma Neuro-2a cells and in primary neurons in a concentration-dependent manner. Absence of FAAH inhibition was demonstrated in different assay systems, including recombinant FAAH, cell lysates, and brain homogenates. Moreover, WOBE437 retained activity in FAAH-deficient HMC-1 human mast cells.^32^

Since its discovery, WOBE437 has been investigated in several animal models where modulating endocannabinoid signaling can be beneficial. In BALB/c mice, WOBE437 was orally bioavailable and induced CB_1_R-dependent anxiolytic, anti-inflammatory, and analgesic effects.^33^ In C57BL/6 (C57) mice, WOBE437 as well as FABP5 inhibitor SBFI-26 were able to lower intraocular pressure in a CB_1_R-dependent manner.^34^ In a mouse model of multiple sclerosis, WOBE437 significantly reduced the severity of the disease and accelerated recovery through CB_1_R (and CB_2_R)-dependent mechanisms.^35^ Although the pharmacological properties of WOBE437 show promise for ECS-directed therapeutics, the mechanism of action remains largely unknown.

To identify targets of WOBE437, photoaffinity-based protein profiling (AfBPP) may be exploited.^36,37^ AfBPP makes use of bifunctional photoaffinity probes, which consist of a ligand of interest functionalized with a photoreactive group and a bioorthogonal ligation handle. After administration of the probe to intact cells, the photoreactive group is activated by UV light. This leads to the formation of a reactive intermediate that may form a covalent bond with amino acids that interact with the probe. An alkyne group in the probe serves as a ligation handle to introduce reporter groups by copper(I)-catalyzed azide–alkyne cycloaddition (CuAAC) chemistry. A fluorophore-azide can be conjugated to visualize interacting proteins by sodium dodecyl sulfate polyacrylamide gel electrophoresis (SDS-PAGE) and fluorescence scanning, or alternatively, a biotin-azide can be ligated for protein isolation and identification using liquid chromatography–mass spectrometry (LC–MS).^38^ Previously, a photoactivatable WOBE437 derivative was reported.^31^ This derivative, RX-055 (**2**, Figure 1), showed similar activity to WOBE437 and retained activity after UV irradiation in washout experiments, whereas WOBE437 did not. This indicated that WOBE437 binds reversibly to a protein target, which can be irreversibly blocked by RX-055. However, as this probe only contains a photoreactive group, it cannot be used in AfBPP experiments to identify the targeted proteins. Therefore, the aim of the current study was to develop an alternative photoaffinity-click probe (pac)-WOBE (**3**) to map the protein interaction landscape of WOBE437 (Figure 1).

## Results

### WOBE437 Increases Anandamide Uptake by Disrupting NAE Levels

**Figure 2:**
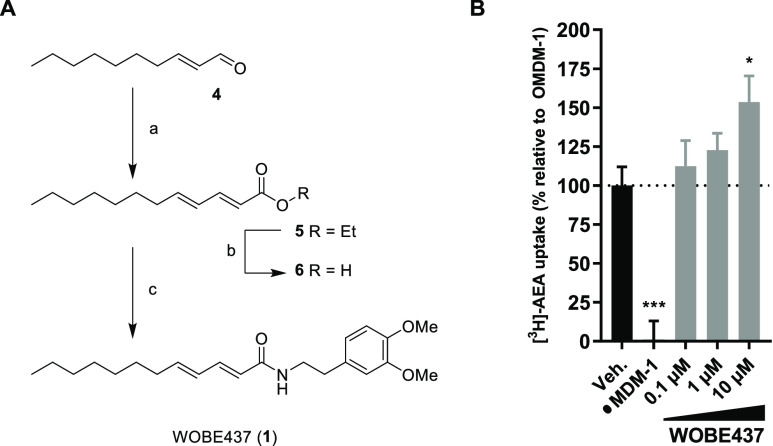
Synthesis and characterization of WOBE437. (A) Reagents and conditions: (a) ethyl 2-(diethoxyphosphoryl)acetate, NaH, 0 °C, then (*E*)-dec-2-enal, −78 °C to rt, 63%; (b) NaOH, 60 °C, quant.; (c) 2-(3,4-dimethoxyphenyl)ethan-1-amine, HOAt, EDC, rt, 78%. (B) Endocannabinoid uptake was assayed in Neuro-2a cells, which were preincubated with OMDM-1 (40 μM) as a positive control or different concentrations of WOBE437 for 10 min. [^3^H]-AEA was added, and cells were incubated for an additional 15 min, washed, and harvested to measure radioactivity. Control experiments were also carried out under the same conditions at 4 °C in order to subtract passive diffusion from active uptake. Data are expressed as means ± SEM of three independent experiments, each performed in triplicate. *p < 0.05; ***p < 0.001 in comparison to vehicle-treated control (dotted line) using one-way ANOVA with Dunnett’s multiple comparisons correction.

First, WOBE437 was synthesized according to a previously reported procedure (Figure 2A).^31^ A Horner–Wadsworth–Emmons reaction with (*E*)-dec-2-enal (**4**) and ethyl 2-(diethoxyphosphoryl)acetate resulted in ester **5**, which was saponified to afford carboxylic acid **6**. A subsequent peptide coupling with 2-(3,4-dimethoxyphenyl)ethan-1-amine gave WOBE437 in 49% yield over three steps. The compound was characterized in a [^3^H]-AEA uptake assay in Neuro-2a cells according to a previously published method.^39^ In brief, Neuro-2a cells were treated with vehicle, WOBE437, or OMDM-1 as a positive control for 10 min in serum-free medium, after which AEA (400 nM) spiked with [^3^H]AEA was added. After 15 min, the cells were thoroughly washed and resuspended in aq. NaOH for measurement in a scintillation counter. Passive uptake at 4 °C was subtracted, and uptake of OMDM-1-treated cells was set as the baseline. In contrast to previous findings,^31^ WOBE437 resulted in a concentration-dependent increase in the uptake of anandamide when compared to the positive control OMDM-1 (Figure 2B).

**Figure 3:**
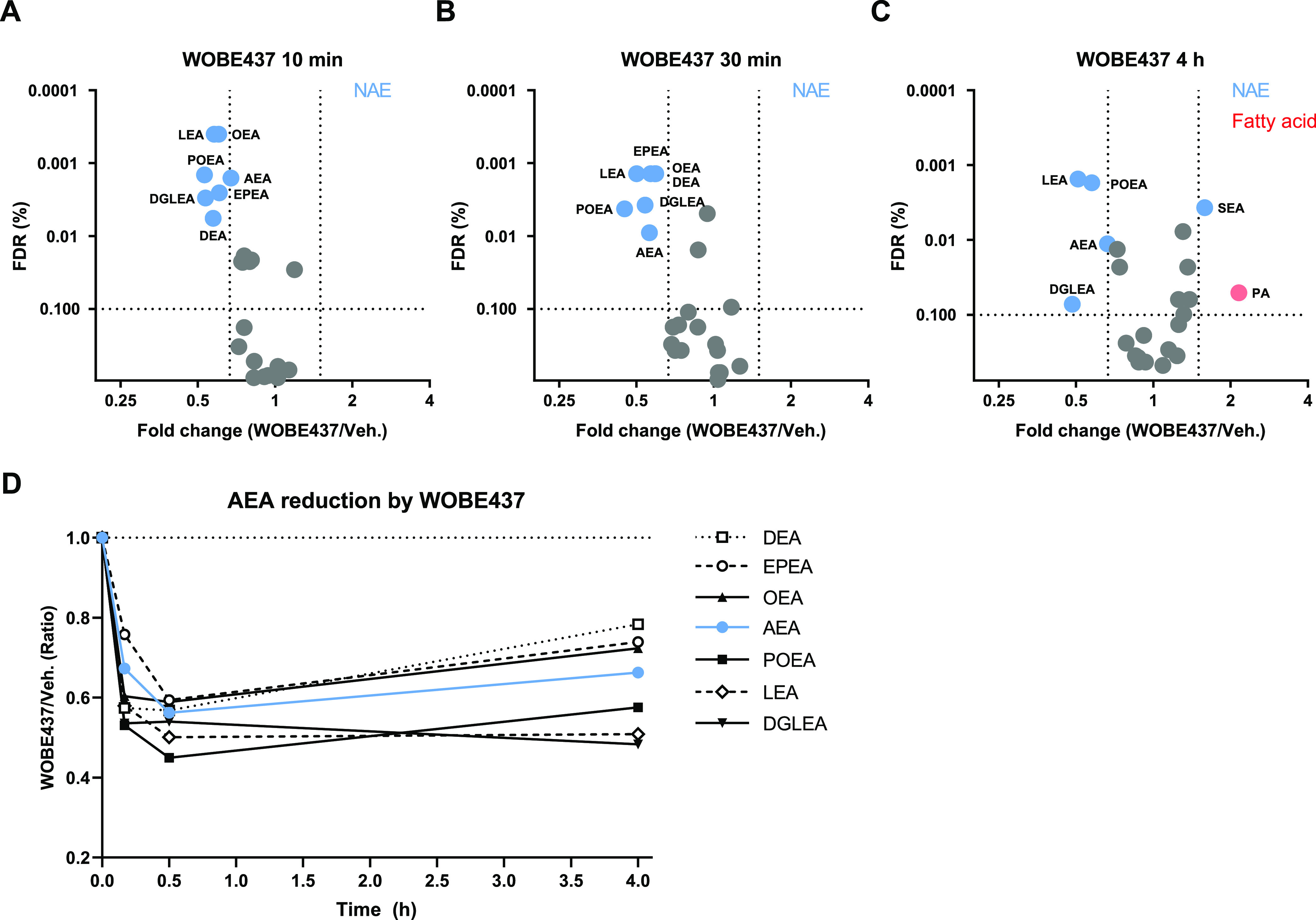
WOBE437 disrupts cellular NAE levels within 10 min of treatment. Neuro-2a cells were treated with 10 μM WOBE437 or vehicle and harvested at the indicated time points to be analyzed by MS-based lipidomics. (A–C) Lipidomic data are presented as a volcano plot, and lipids with a fold-change threshold of ≥1.50 or ≤0.67 and a Benjamini-Hochberg false-discovery rate (FDR) ≤10% following a Student’s *t*-test are represented by colored circles indicating lipid class. (D) Fold-change of altered NAEs is represented as a function of time. The complete list of ratios at 30 min are depicted in Figure S1.

To investigate the cellular effects of WOBE437 in more detail, *N*-acylethanolamines (NAEs), free fatty acids, and monoacylglycerides were measured using a LC-MS-based assay (Table S2). Neuro-2a cells were incubated for different time periods with either WOBE437 or vehicle and washed, and lipids were extracted. Compared to vehicle-treated Neuro-2a cells, WOBE437 induced a time-dependent decrease in all NAE levels, except stearoylethanolamide (SEA), pentadecanoylethanolamide (PDEA), and docosahexaenoylethanolamide (DHEA) (Figures 3, S1). The largest decrease was observed after 30 min. No effect on free fatty acids or monoacylglycerols was found (Figure S2). Previously, the inhibition of AEA uptake by WOBE437 was shown to be dependent on the passage number of Neuro-2a cells,^31^ but no effect of passage number was found in the current study (Figure S2A,B).

The decrease in NAE levels is consistent with increased AEA uptake as the transport is driven by the concentration gradient across the plasma membrane.^21^ To investigate whether the reduction of NAEs was due to inhibition of NAPE-PLD, WOBE437 was tested in a surrogate substrate-based fluorescence assay using purified enzyme (Figure S3A). WOBE437 did not inhibit NAPE-PLD activity nor any other serine hydrolase, as indicated by activity-based protein profiling (Figure S3B).^40^

### Synthesis and Characterization of pac-WOBE (**3**)

**Figure 4:**
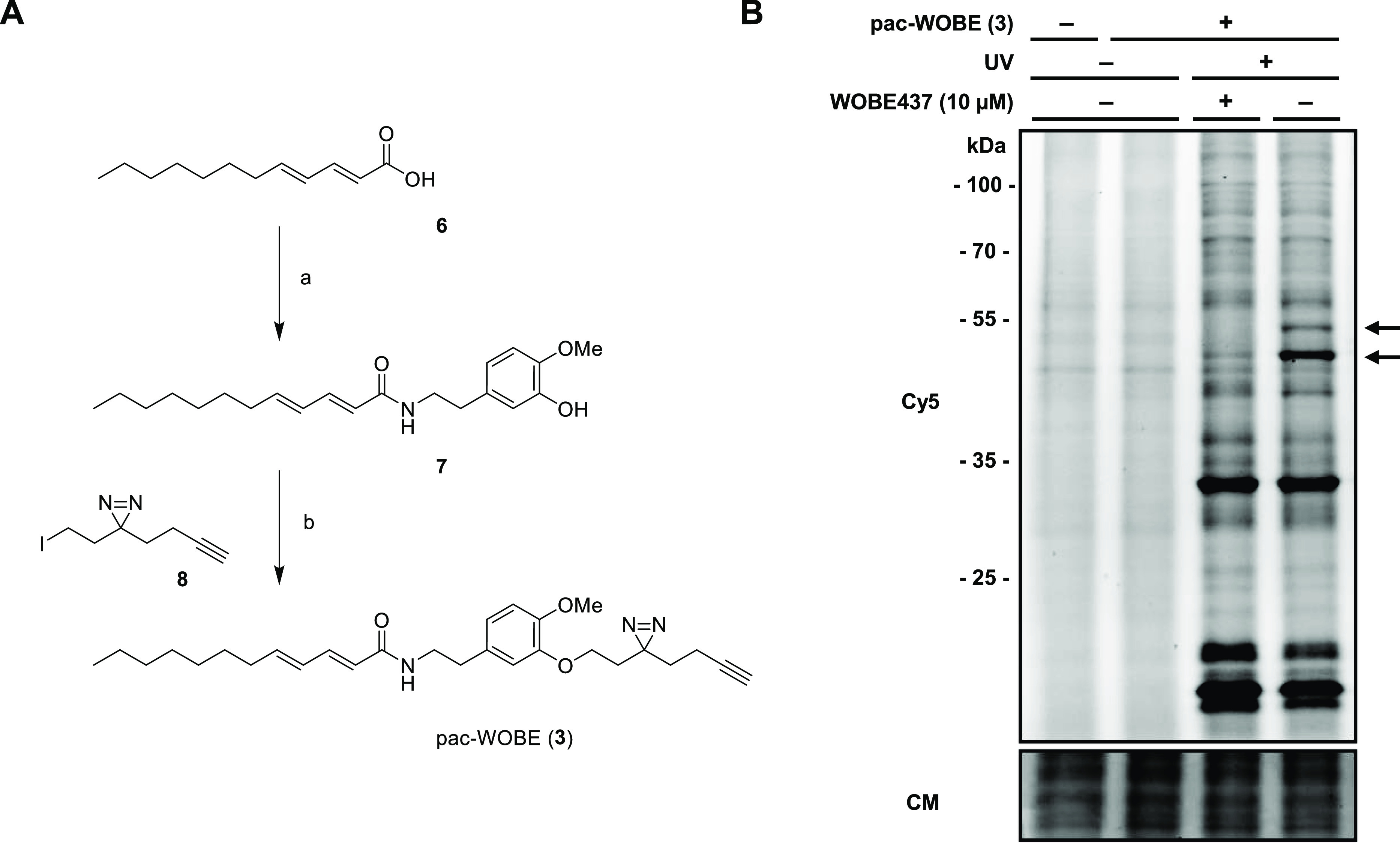
Synthesis and characterization of pac-WOBE (3). (A) Reagents and conditions: (a) 5-(2-aminoethyl)-2-methoxyphenol, HOBt, EDC, 0 °C to rt, 43%; (b) 3-(but-3-yn-1-yl)-3-(2-iodoethyl)-3*H*-diazirine, K_2_CO_3_, 60 °C, 29%. (B) Neuro-2a cells were treated with 10 μM WOBE437 or vehicle and subsequently with 0.1 μM pac-WOBE (**3**) or vehicle, irradiated, and lysed, and proteomes were conjugated to Cy5-N_3_ using CuAAC chemistry and analyzed by SDS-PAGE and in-gel fluorescence scanning. Coomassie served as a protein loading control. Arrows indicate WOBE437-competed targets.

To profile the protein interaction landscape of WOBE437, a photoaffinity probe (**3**) was designed, guided by the reported structure–activity relationship.^31^ A minimalist diazirine and alkyne-containing moiety^41^ was introduced on the phenyl ring of WOBE437 by peptide coupling 5-(2-aminoethyl)-2-methoxyphenol with **6** after which an S_N_2 substitution on 3-(but-3-yn-1-yl)-3-(2-iodoethyl)-3*H*-diazirine afforded pac-WOBE (**3**) in 12% yield over two steps (Figure 4A).

To visualize the protein targets of WOBE437 by gel-based AfBPP, Neuro-2a cells were incubated with **3** and irradiated with UV light (350 nm, 10 min, “UV”) or exposed to ambient light (“no UV”). The cells were harvested and lysed, and the probe-bound proteins were conjugated to Cy5-N_3_ under CuAAC conditions. The protein samples were resolved by SDS-PAGE and visualized by in-gel fluorescence scanning (Figure 4B). This showed that **3** could UV-dependently label several proteins. Pretreatment of the cells with WOBE437 resulted in a reduced labeling intensity of two bands around 50 kDa, which suggested that these proteins specifically interact with WOBE437.

### Identification and Characterization of WOBE437 Targets

**Figure 5:**
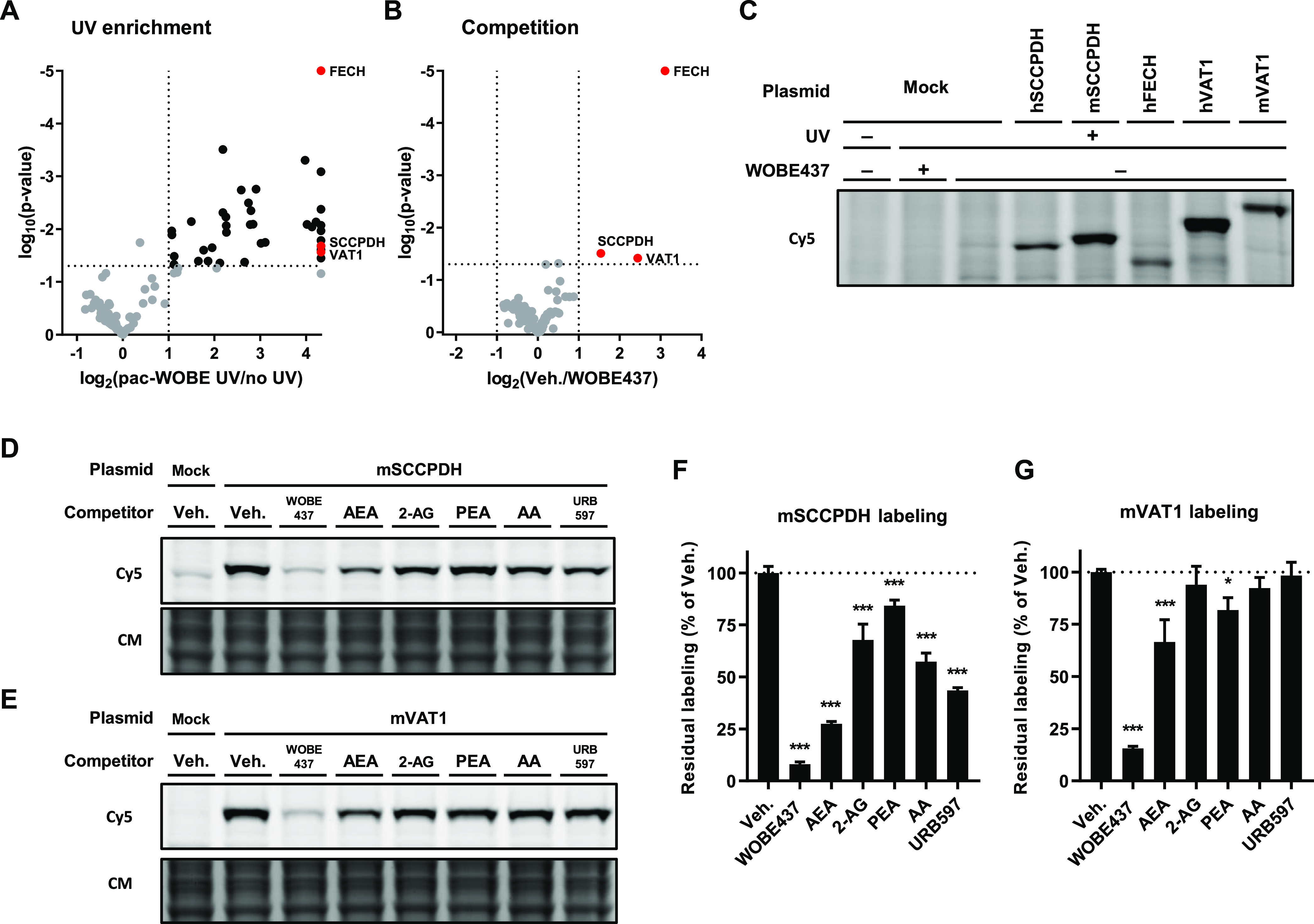
Identification and characterization of WOBE437 targets using pac-WOBE (3). Volcano plot depicting (A) UV enrichment and (B) WOBE437 competition of proteins labeled by *in situ* AfBPP in Neuro-2a cells using 1 μM pac-WOBE (3). UV enrichment is capped at 20-fold, *p*-value at 0.00001. A complete list of targets is available in Table S4. (C) Gel-based AfBPP profiling of overexpressing HEK-293-T cells using 0.1 μM pac-WOBE (3). (D,E) Representative gels of competition of 0.1 μM pac-WOBE (3) labeling of overexpressing HEK-293-T cells by 10 μM of the indicated compound. (F,G) Quantified residual labeling of indicated protein by 0.1 μM pac-WOBE (**3**) after preincubation with the indicated compound. Fluorescent signal was normalized to quantified Coomassie signal. Data represent means ± SD of three biological replicates. *p < 0.05; ***p < 0.001 in comparison to vehicle-treated control (dotted line) using one-way ANOVA with Dunnett’s multiple comparisons correction.

Next, a label-free chemical proteomics experiment was performed to identify the WOBE437-interacting proteins.^42^ Neuro-2a cells were pretreated with WOBE437 (10 μM) or vehicle, after which they were incubated with 0.1 or 1.0 μM pac-WOBE (**3**) with or without UV exposure. Cells were lysed and treated with biotin-N_3_ under CuAAC conditions. Probe-bound proteins were enriched using avidin-coated agarose beads, digested by trypsin, and analyzed by LC–MS/MS. Proteins displaying >2-fold UV enrichment with a *p*-value <0.05 were designated as pac-WOBE-interacting targets. This afforded 8 and 39 significantly UV-enriched targets for the two probe concentrations, respectively (Figures 5A, S4A), of which none could be outcompeted by AEA reuptake inhibitor VDM11 or FAAH inhibitor URB597. Three of these probe targets [saccharopine dehydrogenase-like oxidoreductase (SCCPDH), vesicle amine transport 1 (VAT1) and ferrochelatase (FECH)] could be outcompeted by preincubation with WOBE437 (Figure 5B, S4A).

Mouse and human orthologues of these three targets were recombinantly expressed in HEK-293-T cells, and target engagement with AEA was investigated using gel-based AfBPP (Figure 5C). AEA engaged in a dose-dependent manner with SCCPDH and VAT1 (Figure S5A,B). AEA did not compete with pac-WOBE (**3**) labeling of FECH, a mitochondrial enzyme extensively studied for its role in heme biosynthesis, and a common off-target of kinase inhibitors^43^ and lipid probes.^44^

In view of these results, further experiments were conducted with mouse SCCPDH and mouse VAT1. The latter has previously been shown to be involved in lipid binding and transport.^45,46^ To investigate whether SCCPDH and VAT1 were selective for WOBE437 and AEA, competitive AfBPP was performed with a selection of other closely related lipids, such as 2-arachidonoylglycerol (2-AG), *N*-palmitoylethanolamine (PEA), arachidonic acid (AA), and the FAAH inhibitor URB597 (Figure 5D–G). WOBE437 was the most potent competitor of mSCCPDH labeling followed by AEA > URB597 > AA > 2-AG > PEA. Mouse VAT1 was much more selective since its labeling was only significantly inhibited by WOBE437, AEA, and PEA (Figure 5D–G).

**Figure 6:**
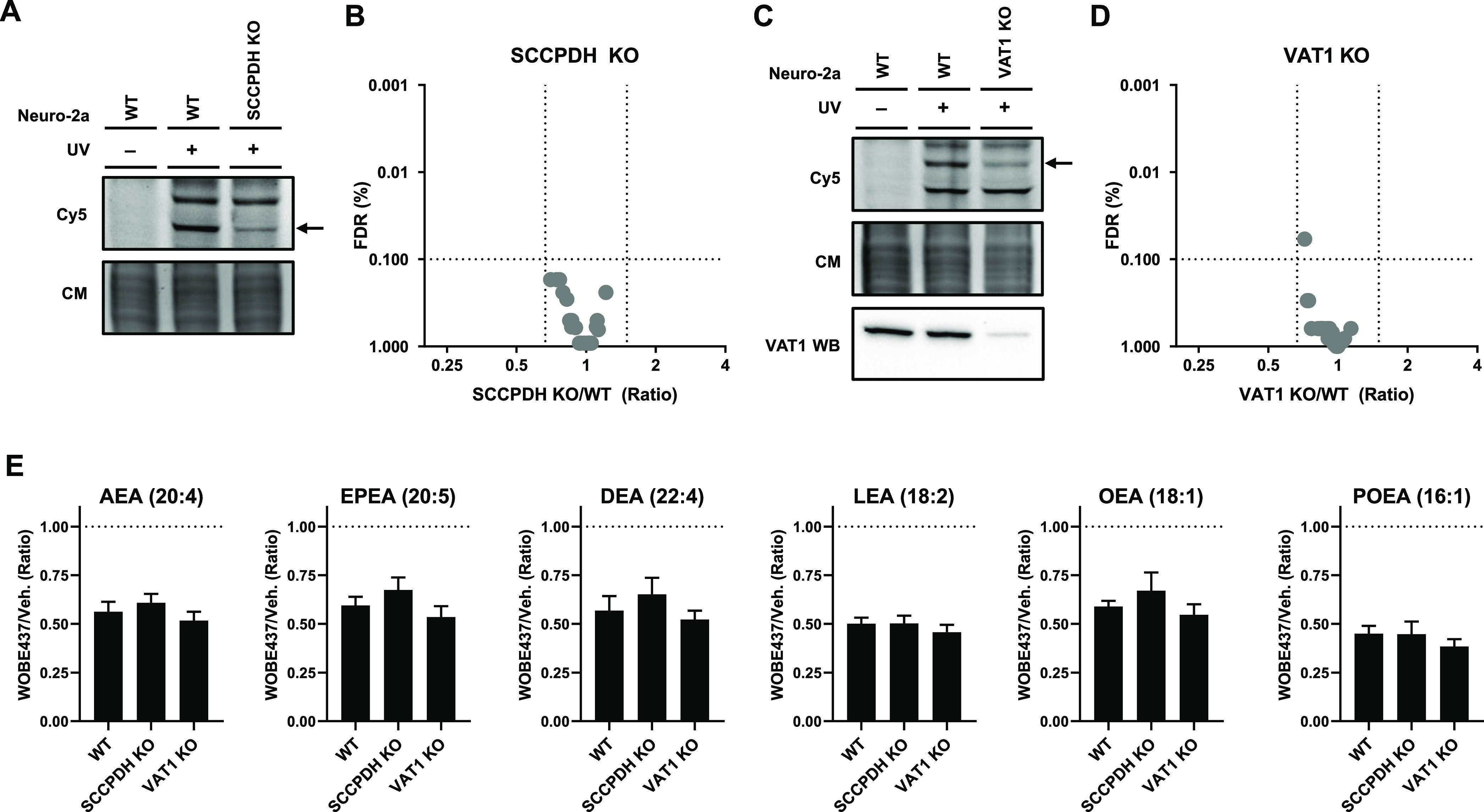
Partial SCCPDH and VAT1 knockouts were generated by CRISPR-Cas9. (A) SCCPDH and (C) VAT1 KO Neuro-2a lines were generated and checked by gel-based AfBPP using 0.1 μM pac-WOBE (3) for residual expression. VAT1 protein was tested by VAT1 Western blot. Coomassie served as a protein loading control. Lipid levels were tested by lipidomics on (B) SCCPDH KO and (D) VAT1 KO cells and compared to WT cells. Further characterization and the complete list of ratios are depicted in Figures S6 and S7. (E) Neuro-2a cells were treated with 10 μM WOBE437 or vehicle for 30 min and harvested to be analyzed by MS-based lipidomics. Lipid levels are displayed as ratio against the same type of cells treated with vehicle. Data represent means ± SEM (*n* = 4). One-way ANOVA with Dunnett’s multiple comparisons correction: not significant when compared to WT.

Next, a genetic approach was used to investigate whether SCCPDH or VAT1 is responsible for WOBE437-induced decrease in NAE levels in Neuro-2a cells since no selective SCCPDH or VAT1 inhibitors are available. Notably, single cell heterogeneity prevented the unequivocal analysis of single cell clone knockouts.^47^ Therefore, disruption of SCCPDH and VAT1 genes was achieved by three consecutive rounds of transfection of Cas9 and single-guide RNAs in Neuro-2a cell populations. SCCPDH and VAT1 expressions in these cell populations were significantly, albeit not completely, decreased as determined by gel-based AfBPP and Western blot for VAT1 (Figure 6A,C). The residual expressions of SCCPDH and VAT1 can be explained by an imperfect transfection efficiency and by insertion or deletion of a full codon upon Cas9-mediated DNA modification, preventing the frameshift that generally results in an early STOP-codon. Next, the cellular NAE levels of these genetically modified Neuro-2a populations were determined using LC–MS and serine hydrolase activity by ABPP. No change in NAE levels or serine hydrolase activity was observed for these knockdown populations compared to wild-type (WT) cells (Figures 6B,D and S6,S7). Notably, WOBE437 was still able to significantly reduce NAE levels in these genetically modified cells (Figure 6E). This indicated that targets other than SCCPDH or VAT1 are responsible for the WOBE437-mediated reduction in NAE levels.

## Conclusions

WOBE437 has been reported as an AEA-uptake inhibitor that shows various *in vivo* effects that are consistent with direct CB_1_R activation by elevated extracellular AEA levels. However, the molecular target of WOBE437 remains unknown. In this study, a photoaffinity-based approach was employed to identify the protein targets of WOBE437 in Neuro-2a cells. Surprisingly, WOBE437 increased AEA uptake and decreased endogenous NAE levels. Although the WOBE437-induced time-dependent decrease in endogenous AEA levels appears in keeping with the increased AEA uptake in Neuro-2a cells, it is unclear why the current results are in contrast to the previous findings.^31^ Differences in the experimental protocol of AEA-uptake experiments and/or heterogeneity of Neuro-2a cells may be contributing factors as washing steps, addition of lipid carrier BSA, or including control experiments at reduced temperatures have previously been shown to affect results of such assays. However, it should be noted that the positive control OMDM-1 did reduce AEA uptake in Neuro-2a cells under the same conditions. At the very least, our findings suggest that OMDM-1 and WOBE437 have different molecular modes of action, although both compounds are reported to act primarily as AEA uptake inhibitors.

AfBPP using a WOBE437-based photoaffinity probe identified SCCPHD, VAT1, and FECH as WOBE437-interacting proteins in Neuro-2a cells. Competitive gel-based AfBPP demonstrated that SCCPHD and VAT1, but not FECH, could bind AEA preferentially over related lipids. CRISPR/Cas9 knockouts of SCCPDH and VAT1, however, indicated that these proteins were not responsible for the WOBE437-induced reduction in endogenous NAE levels in Neuro-2a cells. It remains to be investigated whether FECH is involved in this process. Regardless of the exact mechanism of action of WOBE437, the current study identified SCCPDH, VAT1, and FECH as off-targets of WOBE437, which may confound the interpretation of the biological effects obtained with this compound.

## Materials and Methods

### Cell Culture

#### General Cell Culture

Neuro-2a and HEK-293-T cells were maintained at 37 °C under 7% CO_2_ atmosphere in DMEM (Sigma-Aldrich, HEK-293-T: D6546, Neuro-2a: D1145) containing stable glutamine, 10% (v/v) Newborn Calf Serum (Thermo Fisher), and penicillin and streptomycin (200 μg/mL of each, Duchefa). Cells were passaged two times per week at 80–90% confluence by resuspending them in fresh medium. Cell lines were purchased from ATCC and were tested regularly for mycoplasma contamination. Cultures were maintained for 2–3 months before being disposed. SCCPDH/VAT1/FECH-overexpressing Neuro-2a or HEK-293-T cells were produced by seeding resuspended cells on 12-well plates (4.0 × 10^4^ cells/cm^2^) 24 h prior to transfection. The culture medium was then aspirated and replaced with 400 μL of fresh medium. A mixture of polyethylenimine [PEI, Neuro-2a: 5:1 (m/m), HEK-293-T: 3:1 (m/m)] and plasmid DNA (0.625 μg/well) was diluted in serum-free medium (100 μL) and incubated for 15 min at rt. Cells were transfected by addition of the PEI/DNA mixture to the cells. After 24 h, the medium was aspirated, and fresh, complete medium was added. Cells were used 48 h post transfection.

### Photoaffinity-Based Protein Profiling

#### Gel-Based AfBPP

For gel-based profiling, transfected HEK-293-T or Neuro-2a (WT or KO) on 12-well plates were treated with the probe as follows: Growth medium was aspirated, a solution of indicated competitor (2X, 10 μM final) or vehicle in serum-free DMEM supplemented with 0.1% (w/v) delipidated BSA (0.5 mL) was added, and the cells were incubated for 30 min at 37 °C. Then, a solution of pac-WOBE (**3**, 2X, 100 nM final) in serum-free DMEM supplemented with 0.1% (w/v) delipidated BSA (0.5 mL) was added, and the cells were incubated for 30 min at 37 °C. The medium was aspirated and replaced with 1 mL of ice-cold DPBS, and the cells were irradiated using a Caprobox (10 min, 4 °C, 350 nm, “UV”) or exposed to ambient light (10 min, 4 °C, “No UV”). The cells were harvested by pipetting and pelleted by centrifugation (1000*g*, 10 min, 4 °C). The supernatant was removed, and the cells were lysed by resuspension in lysis buffer (250 mM sucrose, 20 mM HEPES pH 7.5, 1 mM MgCl_2_, 1X protease inhibitor cocktail (Roche), 25 U/mL benzonase) and sonication in a bath sonicator (0 °C, 5 min). Protein concentration was measured by Qubit assay (Invitrogen), and the samples were adjusted to 1.5 mg/mL and a volume of 100 μL, after which the samples were treated with 10.4 μL of click mix (5.5 μL of aq 25 mM CuSO_4_, 3.25 μL of aq 250 mM NaAsc, 1.1 μL of 25 mM THPTA in DMSO, and 0.55 μL of 0.9 mM Cy5-N_3_ in DMSO) and left at rt for 1 h. Samples were then quenched by addition of 4× Laemmli buffer, boiled (5 min, 95 °C), and resolved by SDS-PAGE (10% acrylamide gel, ±80 min, 180 V) along with a protein marker (PageRuler Plus, Thermo Fisher). In-gel fluorescence was measured in the Cy3- and Cy5-channels (Chemidoc MP, Bio-Rad), and the gels were subsequently stained with Coomassie and imaged as a loading control for normalization of fluorescence intensity. Band intensities were quantified using Image Lab 6.0.1 (BioRad).

For the VAT-1 Western blot, part of the gel was stained with Coomassie and imaged for loading control. The rest of the gel was transferred to a 0.2 μm polyvinylidene difluoride membrane by Trans-Blot Turbo Transfer system (Bio-Rad). Membranes were washed with TBS (50 mM Tris pH 7.5, 150 mM NaCl) and blocked with 5% (w/v) milk in TBS-T [50 mM Tris pH 7.5, 150 mM NaCl, 0.05% (w/v) Tween-20] for 1 h at rt. Membranes were then washed three times with TBS-T, followed by incubation with primary antibody in 5% (w/v) BSA in TBS-T (VAT1, PA5-43777, Thermo Fisher, 1:1,000, 1 h, rt). Membranes were then washed three times with TBS-T and incubated with secondary goat-anti-rabbit-HRP [sc-2030, Santa Cruz, 1:5,000 in 5% (w/v) milk in TBS-T, 1 h, rt] and then washed three times with TBS-T and once with TBS before developing. Membranes were developed in luminol development solution [10 mL of 1.4 mM luminol in 100 mM Tris pH 8.8 + 100 μL of 6.7 mM *p*-coumaric acid in DMSO + 3 μL of 30% (v/v) H_2_O_2_], and chemiluminescence was detected on ChemiDoc MP (Bio-Rad) in the chemiluminescence channel and colorimetric channel for the protein marker. Images were processed using Image Lab 6.0.1 (BioRad).

### Chemical Proteomics-Based AfBPP

Neuro-2a cells were plated on 6-well plates and grown to near confluency (90%). The supernatant was aspirated; serum-free DMEM supplemented with 0.1% (w/v) delipidated BSA (1 mL) and WOBE-437 or vehicle was added, and the cells were incubated for 30 min at 37 °C. After this period, pac-WOBE (**3**) was added, and the cells were incubated for 30 min at 37 °C. Subsequently, the medium was aspirated and replaced with 1 mL of ice-cold DPBS, and the cells were irradiated using a Caprobox (10 min, 4 °C, 350 nm, “UV”) or exposed to ambient light (10 min, 4 °C, “No UV”). The cells were harvested by pipetting and pelleted by centrifugation (1000*g*, 10 min, 4 °C). The supernatant was removed, and the cells were lysed by resuspension in lysis buffer [250 μL, 250 mM sucrose, 20 mM HEPES pH 7.5, 1 mM MgCl_2_, 1× protease inhibitor cocktail (Roche)] and sonication (Branson Sonifier probe sonicator, 10 × 2 s pulses, 10% amplitude). Protein concentration was measured by the Qubit assay (Invitrogen), and the samples were adjusted using 50 mM HEPES pH 7.5 to a protein concentration of 1.0 mg/mL and a volume of 400 μL. The pulldown experiment was performed as earlier described, with minor adjustments.^42,48^ The lysates (400 μL) were subjected to a click reaction with freshly prepared click mix (43.7 μL per sample: 21.9 μL of aq 25 mM CuSO_4_, 13 μL of aq 250 mM NaAsc, 4.4 μL of 25 mM THPTA in DMSO, and 4.4 μL of 2.25 mM biotin-N_3_ in DMSO) at rt for 1 h. Proteins were precipitated by addition of HEPES buffer (50 μL, 50 mM, pH 7.5), MeOH (666 μL), CHCl_3_ (166 μL), and MilliQ (150 μL), vortexing after each addition. After spinning down (1500*g*, 10 min), the upper and lower layers were aspirated, and the protein pellet was resuspended in MeOH (600 μL) by sonication (Branson Sonifier probe sonicator, 10 × 0.5 s pulses, 10% amplitude). The proteins were spun down (20,000*g*, 5 min), and MeOH was aspirated. The proteins were redissolved in 6 M urea (500 μL) with 25 mM NH_4_HCO_3_ for 15 min, followed by reduction (65 °C, 15 min, 800 rpm shaking) with dithiothreitol (5 μL, 1 M). The samples were allowed to reach rt and proteins were alkylated (30 min) with indole-3-acetic acid (40 μL, 0.5 M) in the dark. 140 μL of SDS (10% w/v) was added, and the samples were spun down (1000*g*, 5 min). They were transferred to 5 mL of PBS containing 50 μL of avidin agarose resin (Pierce, 100 μL of a 50% slurry, prewashed twice with 6 mL of PBS + 0.5% SDS and once with 6 mL of PBS) and incubated for 2 h while rotating. The beads were spun down (2000*g*, 2 min) and washed (3 × PBS + 0.5% SDS, 2 × PBS, 1 × Milli-Q). The beads were resuspended in digestion buffer [250 μL of 100 mM Tris pH 7.8, 100 mM NaCl, 1 mM CaCl_2_, 2% (v/v) acetonitrile and sequencing grade trypsin (Promega, 0.25 μg)] and transferred to low-binding tubes (Sarstedt) and incubated while shaking overnight (16 h, 37 °C, 1000 rpm). Trypsin was quenched with 12.5 μL of formic acid (LC–MS grade), and the beads were filtered off over a Bio-Spin column (BioRad, 400*g*, 5 min), collecting the flow-through in a new 2 mL tube. Samples were added on C18 stagetips^49^ [preconditioned with 50 μL of MeOH, then 50 μL of 0.5% (v/v) formic acid in 80% (v/v) acetonitrile/Milli-Q (solution B), and then 50 μL of 0.5% (v/v) formic acid in Milli-Q (solution A) by centrifugation (600*g*, 2 min)]. The peptides were washed with solution A (100 μL, 800*g*, 3 min) and eluted into new low-binding tubes using solution B (100 μL, 800*g*, 3 min). Samples were concentrated using an Eppendorf speedvac (Eppendorf Concentrator Plus 5301) and redissolved in LC–MS solution [30 μL per sample: 28.5 μL of Milli-Q, 2.85 μL of acetonitrile, 0.095 μL of formic acid, and 600 fmol yeast enolase peptide digest (Waters Corporation, 186002325)].

Measurement of the samples was done on a NanoACQUITY UPLC System linked to a SYNAPT G2-Si high-definition mass spectrometer (waters). The dissolved peptides were run on an analytical column (HSS-T3 C18 1.8 μm, 75 μm × 250 mm, waters) with a concave gradient (5 to 40% acetonitrile in H_2_O with 0.1% formic acid). For the lock mass, [Glu^1^]-fibrinopeptide B was used. Acquisition of mass spectra was done using the UDMS^e^ method. Mass range was set between 50 and 2000 Da with a scan time of 0.6 s in the positive, resolution mode. For the low-energy MS mode, collision energy was set to 4 V in the trap cell. Transfer cell collision energy was ramped using drift-time-specific collision energies for the elevated energy scan. The lock mass was sampled every 30 s. For raw data processing, Progenesis QI for proteomics was used with the following parameters to search the murine proteome from Uniprot (Table S1). Proteins identified by Progenisis QI were filtered to be identified by at least two unique peptides to obtain high-confidence proteins. To determine significantly UV-enriched probe targets, the ratio of LFQ values of three replicates was calculated, as well as a Student’s *t*-test between the UV- and no UV-treated samples. To determine the competition, this process was repeated, requiring a ratio of at least 2 between competitor- and vehicle-treated samples.

### AEA Uptake Assay

The uptake of AEA was measured in Neuro-2a cells (seeded in triplicate in 12-well plates) according to the literature protocol with minor modifications.^39^ Neuro-2a cells were preincubated in serum-free medium with OMDM-1 (40 μM) for 15 min or different concentrations of WOBE437 (0.1, 1, and 10 μM) or vehicle for 10 min by adding the substance directly to the incubation medium. Then, the cells were incubated with AEA (400 nM) supplemented with [arachidonoyl-5,6,8,9,11,12,14,15-^3^H]AEA (30,000 cpm, ARC, St. Louis, MO, USA) at 37 °C for 15 min. The medium was then aspirated, and the cells were washed three times with PBS supplemented with 1% (w/v) BSA (1 mL) and resuspended in aq NaOH (0.5 M, 0.5 mL) and measured in a scintillation counter. Control experiments were also carried out at 4 °C in order to subtract passive diffusion from active uptake.

### Lipidomics

Neuro-2a (WT or KO) cells were suspended and counted, and 2.5 × 10^6^ cells were seeded in 6 cm dishes (Sarstedt) and allowed to recover for 48 h. Cells were then washed with DBPS (2 mL), and treatment was initiated by addition of 2 mL of serum-free DMEM with 0.1% (w/v) delipidated BSA with indicated concentration of WOBE437 or vehicle (0.1% EtOH) for indicated times at 37 °C and 7% CO_2_. After incubation, the treatment medium was aspirated, and the cells were washed with DPBS (2 mL) and then harvested on ice with ice-cold DPBS, of which a portion was reserved for normalization using protein concentration after lysis using probe sonication (Branson Sonifier probe sonicator, 5 s, 30% amplitude). Cells for lipidomic analysis were spun down (1000*g*, 5 min, 4 °C), the supernatant was aspirated, and the cells were snap-frozen. Samples were thawed on ice and spiked with 10 μL of deuterium labeled internal standard mix (Table S2), vortexed, and incubated for 5 min on ice. Subsequently, NaCl [0.5% (w/v), 100 μL] and NH_4_Ac (0.1 M, pH 4, 100 μL) were added. Ice-cold methyl *tert*-butyl ether (MTBE) (HPLC grade, 1 mL) was added, and the tubes were thoroughly mixed for 7 min using a bullet blender blue (Next advance Inc., Averill park, NY, USA) at speed 8, followed by a centrifugation step (16,000*g*, 11 min, 4 °C). Next, 925 μL of the upper MTBE layer was transferred into a clean 1.5 mL Safe-Lock Eppendorf tube. Samples were concentrated in a speedvac (Eppendorf, ±45 min, 30 °C) and reconstituted in ACN/Milli-Q (30 μL, 90:10 v/v) by thorough mixing for 4 min, followed by a centrifugation step (10,000*g*, 4 min, 4 °C), and transferred to an LC–MS vial (9 mm, 1.5 mL, amber screw vial, KG 090188, Screening Devices) with insert (0.1 mL, tear drop with plastic spring, ME 060232, Screening devices). 5 μL of each sample was injected into the LC–MS system.

Targeted lipidomics was performed on a panel of 23 lipids consisting of endocannabinoids, related NAEs, and free fatty acids (Table S2). Lipidomics measurements were performed on an Acquity UPLC I class binary solvent manager pump linked to a tandem quadrupole mass spectrometer (Waters Corporation). Lipids were separated using a Acquity HSS T3 column (2.1 × 100 mm, 1.8 μm) kept at 45 °C. The mobile phases consisted of a solution of 2 mM ammonium formate and 10 mM formic acid in Milli-Q as phase A and acetonitrile as phase B. Flow rate was set to 0.55 mL/min, and lipids were separated using an initial gradient of 55% B held for 2 min, which was linearly increased to 100% B over 6 min and held for 2 min. The column was equilibrated with 55% B held for 2 min before each run. For lipid quantification, electrospray ionization-mass spectroscopy and selective multiple reaction mode (MRM) were used. MRM transitions for each lipid were optimized using synthetic references and internal standards (Table S2). Peak area integration was performed with MassLynx 4.1 software (Waters Corporation). The quantified peak areas were divided over the peak areas of the corresponding internal standards to obtain response ratios, which were translated to absolute concentrations using their respective calibration curves. Concentrations were then normalized to the amount of protein in the sample as determined by the Bradford assay.

The mass spectrometry proteomics data have been deposited to the ProteomeXchange Consortium via the PRIDE^50^ partner repository with the dataset identifier PXD031488.

## Supporting Information Available

Methods and materials used and additional figures (PDF)
Complete list of identified proteins from photoaffinity experiments (XLSX)
