# Supplementary material for: Chemical Proteomics Reveals Off-Targets of the Anandamide Reuptake Inhibitor WOBE437
Source: ACS Chem Biol. 2022 Apr 28;17(5):1174–83. doi: 10.1021/acschembio.2c00122 (PMC9127799; doi:10.1021/acschembio.2c00122)
Supplement: Supplementary file 1 — cb2c00122_si_001.pdf [file cb2c00122_si_001.pdf]

Supporting information for

Chemical proteomics reveals off-targets of the anandamide reuptake inhibitor WOBE437

Berend Gagestein[1], Anna F. Stevens[1], Domenico Fazio[2], Bogdan I. Florea[4], Tom van der Wel[1], Alexander T. Bakker[1], Filomena Fezza[3], Hans den Dulk[1], Herman S. Overkleeft[4], Mauro Maccarrone[2,5*], Mario van der Stelt[1*]

[1]Department of Molecular Physiology, Leiden Institute of Chemistry, Leiden University, Einsteinweg 55, Leiden 2333 CC, The Netherlands; [2]European Center for Brain Research/IRCCS Santa Lucia Foundation, Via del Fosso di Fiorano 64, 00143 Rome, Italy; [3]Department of Experimental Medicine, Tor Vergata University of Rome, Via Montpellier 1, 00121 Rome, Italy; [4]Bio-Organic Synthesis, Leiden Institute of Chemistry, Leiden University, Einsteinweg 55, Leiden 2333 CC, The Netherlands; [5]Department of Biotechnological and Applied Clinical Sciences, University of L'Aquila, Via Vetoio snc, 67100 L'Aquila, Italy.

[*]Shared senior and corresponding authors. E-mail: mauro.maccarrone@univaq.it; m.van.der.stelt@chem.leidenuniv.nl

Supplementary figures and tables

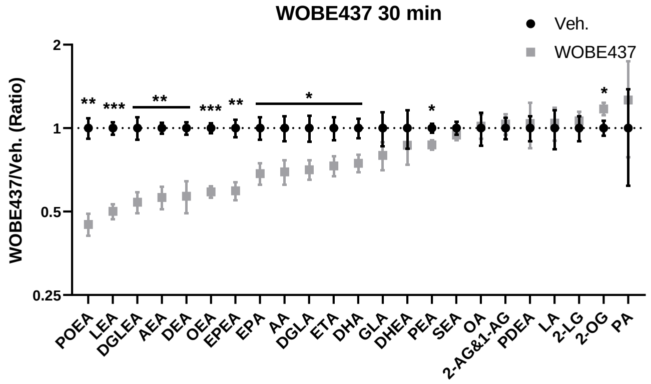

Figure S1. Lipid levels of Neuro-2a cells after 30 min of WOBE437 treatment corresponding to Figure 3. Neuro-2a cells were treated with 10 µM WOBE437 or vehicle and harvested after 30 min to be analyzed by MS-based lipidomics. Lipid levels are relative to vehicle-treated control. Data represent means ± SEM (n = 4), t-test with Benjamini-Hochberg correction: * q <0.05, ** q <0.01, *** q <0.001.

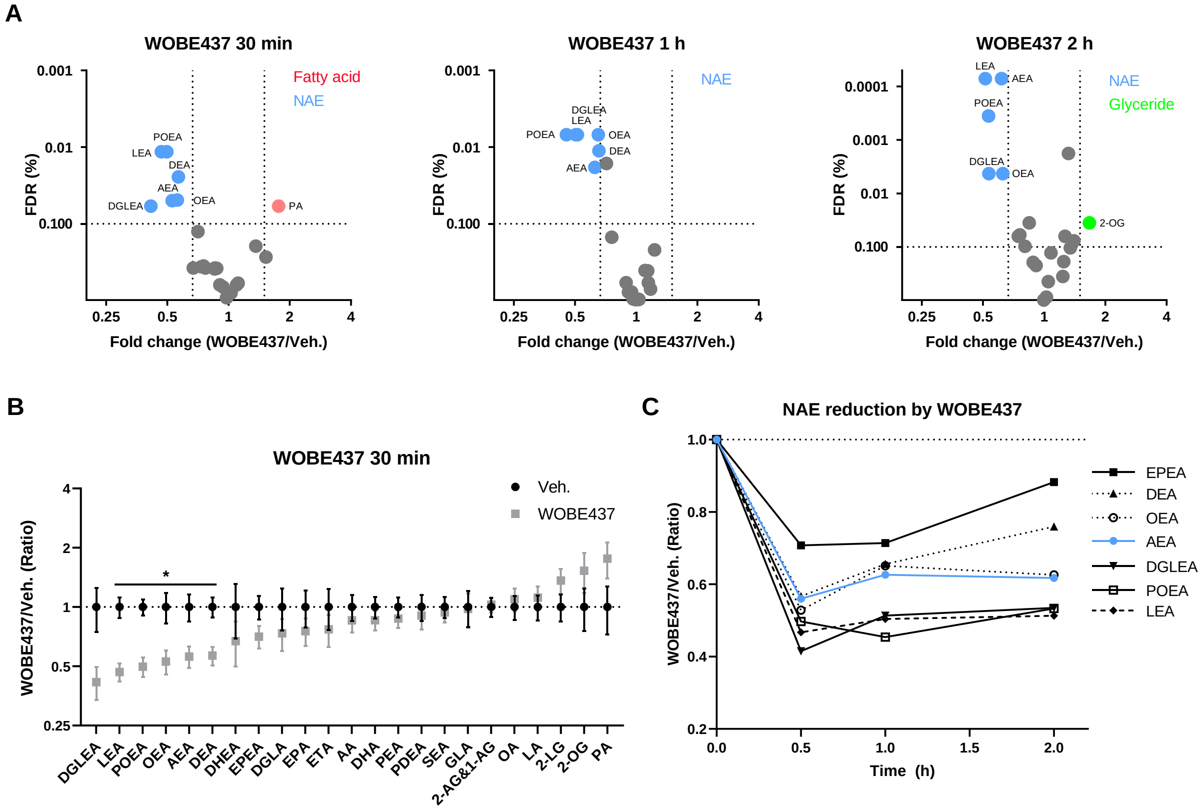

Figure S2. NAE disruption of WOBE437 on different passage of Neuro-2a. Neuro-2a cells kept in culture for over 2.5 months were treated with 10 µM WOBE437 or vehicle and harvested at the indicated time points to be analyzed by MS-based lipidomics. **(A)** Lipidomic data are presented as a volcano plot and lipids with a fold-change threshold of ≥1.50 or ≤0.67 and a Benjamini-Hochberg false-discovery rate (FDR) ≤10% are represented by colored circles indicating lipid class (n = 4). **(B)** All ratios of measured lipids after 30 min of WOBE437 treatment. Lipid levels are relative to vehicle-treated control. Data represent means ± SEM (n = 4), t-test with Benjamini-Hochberg correction: * q <0.05. **(C)** Selected NAE ratios over time after WOBE437 incubation.

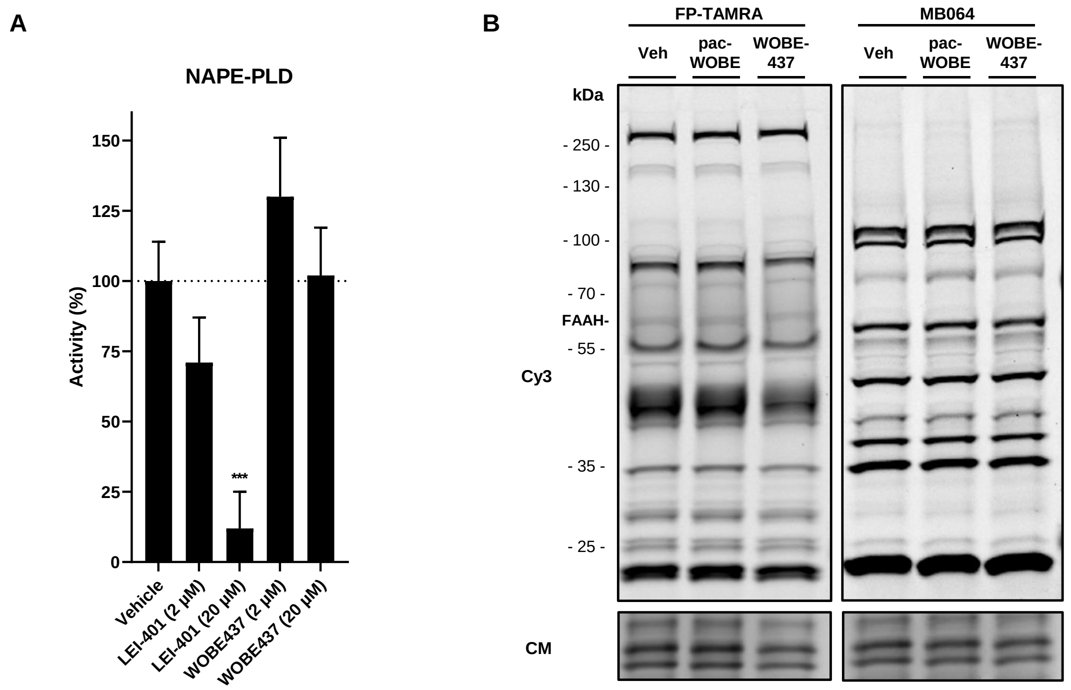

Figure S3. NAPE-PLD activity assay and serine hydrolase ABPP. (A) Activity of recombinant human NAPE-PLD as tested in a PED6 surrogate substrate assay using LEI-401 as positive control.[1] Data represent means ± SD (n = 4), one-way ANOVA with Dunnett's multiple comparisons correction: *** p <0.001 in comparison to vehicle-treated control (Dotted line). **(B)** Neuro-2a lysate was preincubated with 10 μM of indicated compound or vehicle followed by FP-TAMRA or MB064 after which the labeled proteins were resolved by SDS-PAGE and in-gel fluorescence was detected. Coomassie served as a protein loading control.

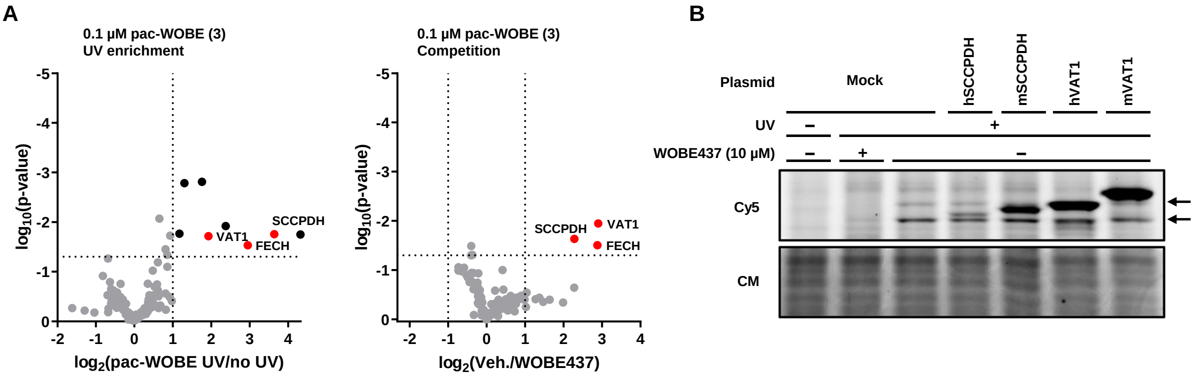

Figure S4. Pulldown and overexpression of pac-WOBE (3) targets in Neuro-2a. (A) Volcano plots of pulldown experiment using 0.1 μM pac-WOBE (**3**) and 10 μM WOBE437 corresponding to Figure 5, Table S4. **(B)** Neuro-2a cells were transfected with indicated plasmid and treated with 10 μM WOBE437 or vehicle and subsequently with 0.1 μM pac-WOBE (**3**), irradiated, lysed and proteomes were conjugated to Cy5-N_3 using CuAAC chemistry and analyzed by SDS-PAGE and in-gel fluorescence scanning. Coomassie served as a protein loading control. Arrows indicate endogenous WOBE437 targets.

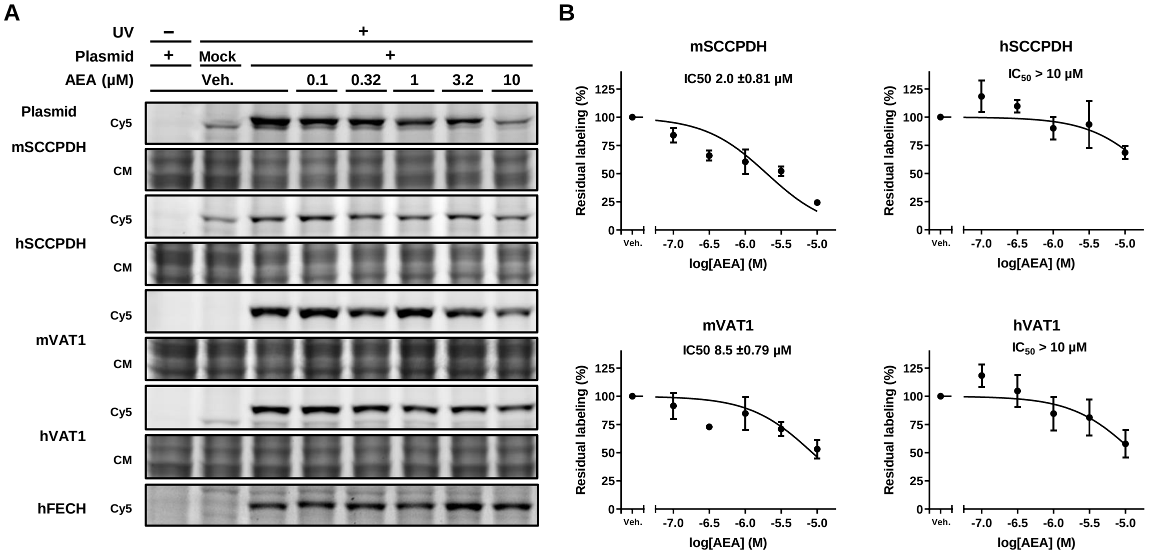

Figure S5. Characterization of WOBE437 targets using pac-WOBE (3). (A) Representative gels of AEA competition of 0.1 µM pac-WOBE (**3**) labeling of overexpressing HEK-293-T cells. **(B)** Quantified residual labeling of indicated protein by 0.1 µM pac-WOBE (**3**) after AEA preincubation at indicated concentration. Fluorescent signal was quantified, normalized by integrated Coomassie signal and expressed as remaining labeling compared to vehicle. Data represent means ± SD of three separate experiments and IC_{50} ± SD.

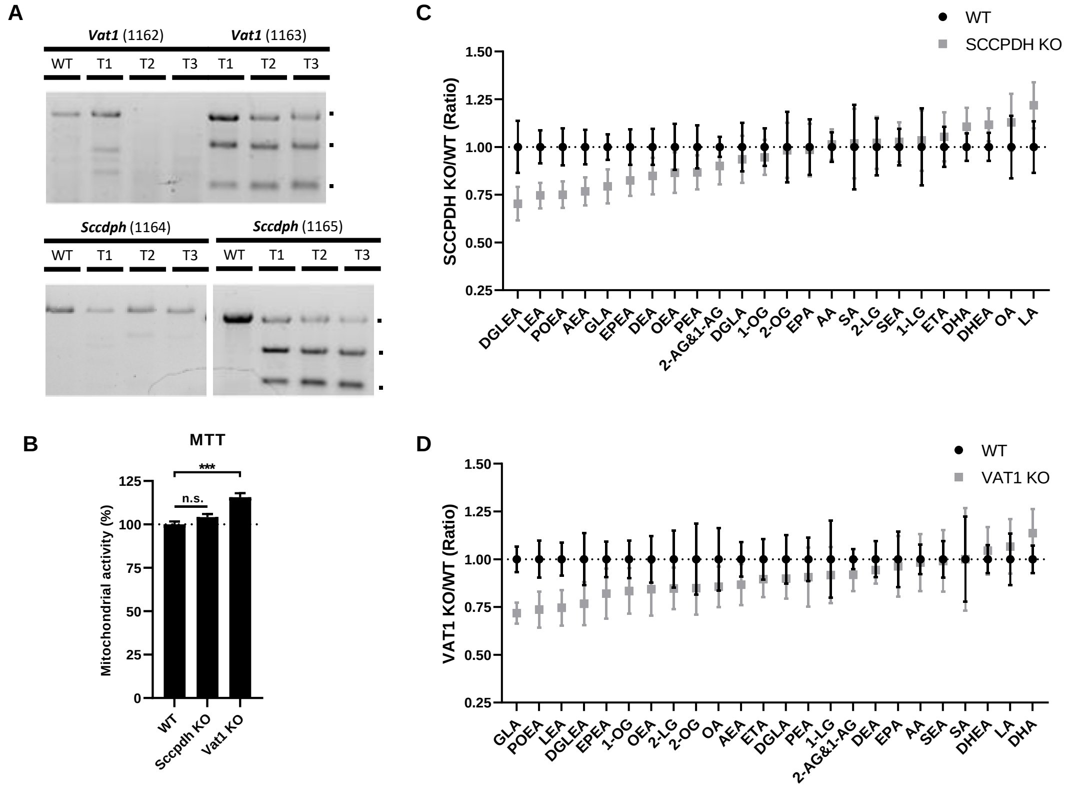

Figure S6. Characterization of CRISPR/Cas9-mediated knockouts in Neuro-2a. (A) Knockout populations were generated by three sequential transfections (T1-T3) with Cas9 and two different separate guides for each target. Knockdown efficiency was determined by a T7E1 assay on genomic DNA, which was analyzed after each round of transfection for *Vat1* and *Sccdph* knockouts. **(B)** $2.0x10^5$ Neuro-2a cells were plated and after 24 h checked for mitochondrial activity by MTT assay. Values shown are mean mitochondrial activity relative to WT Neuro-2a ± SEM (n = 6), one-way ANOVA with Dunnett's multiple comparisons correction: *** p <0.001 in comparison to WT. **(C)** SCCPDH KO and **(D)** VAT1 KO lipid ratios corresponding to Figure 6. Lipid levels relative to WT Neuro-2a. Data represent means ± SEM (n = 4), t-test with Benjamini-Hochberg correction: not significant.

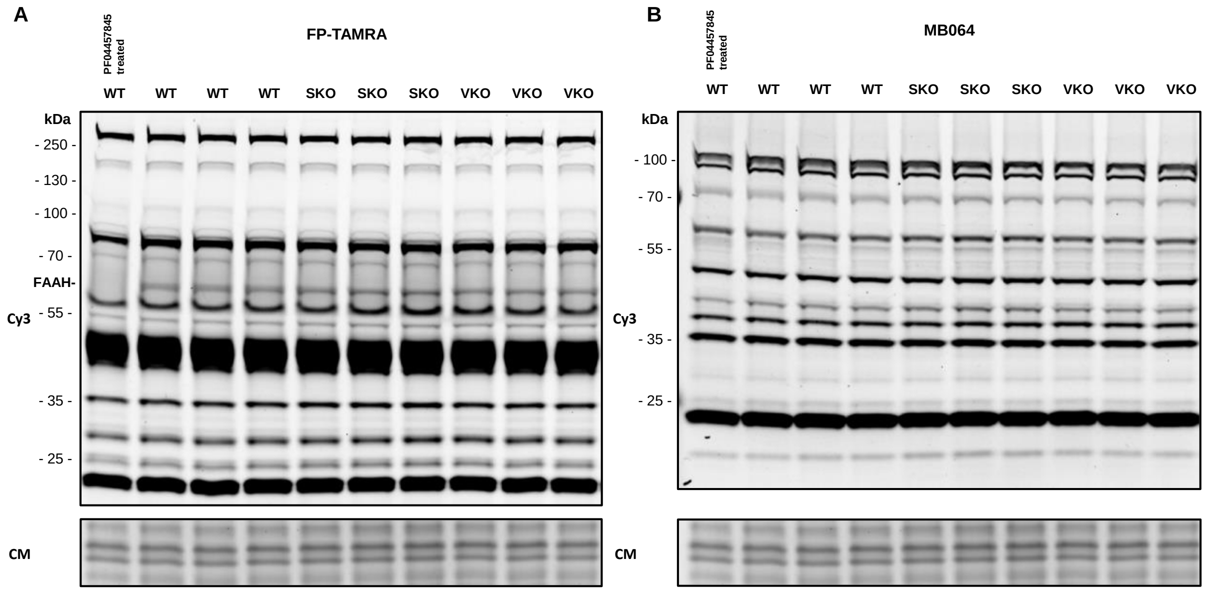

Figure S7. SCCPDH (SKO) or VAT1 (VKO) knockdown does not affect serine hydrolases labeled by FP-TAMRA or MB064. Neuro-2a serine hydrolases were labeled with **(A)** FP-TAMRA or **(B)** MB064 in triplicate and the labeled proteins were resolved by SDS-PAGE and in-gel fluorescence was detected. Coomassie served as a protein loading control.

Figure 4B - Cy5

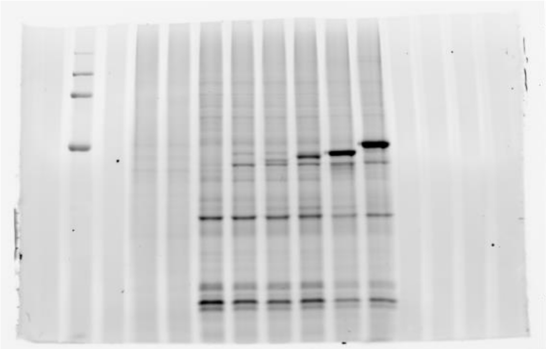

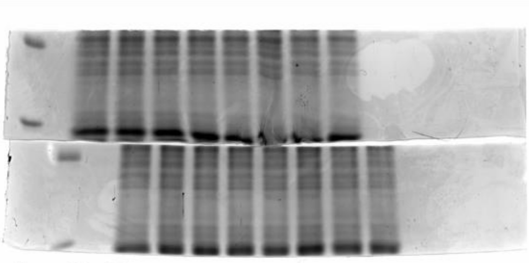

Figure 4B - CM

Figure S8. Uncropped gels for Figure 4.

Figure 5C - Cy5

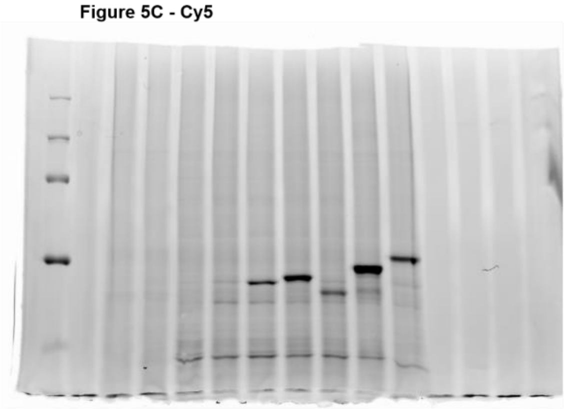

Figure 5D - Cy5

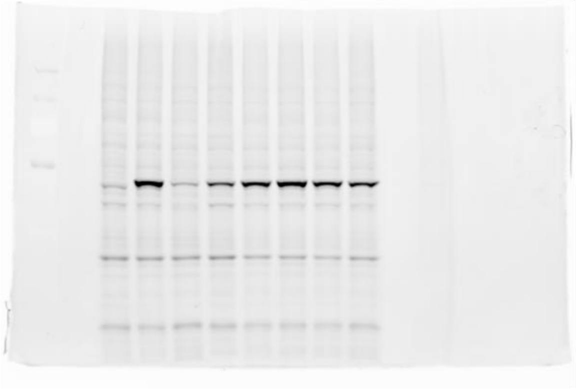

Figure 5E - Cy5

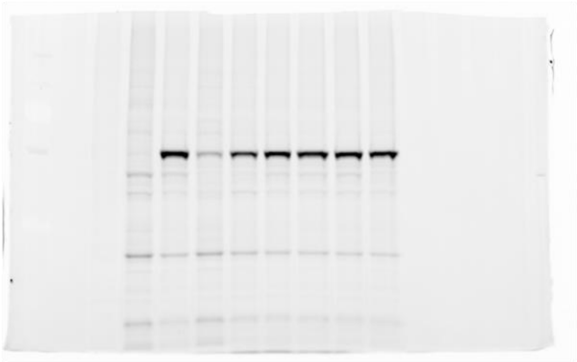

Figure 5D - CM

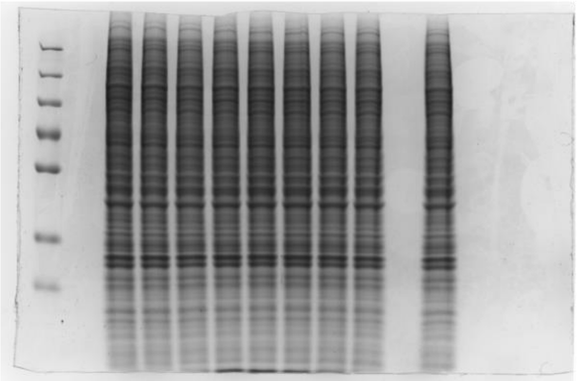

Figure 5E - CM

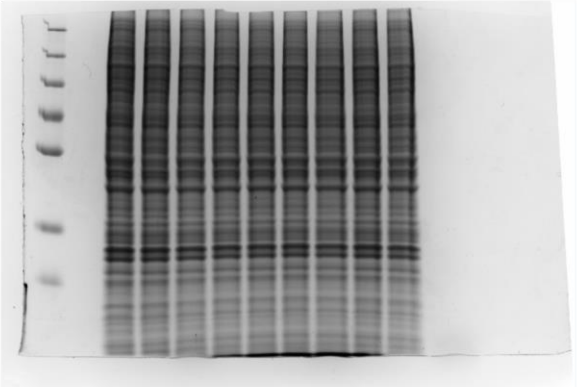

Figure S9. Uncropped gels for Figure 5.

Figure 6A - Cy5

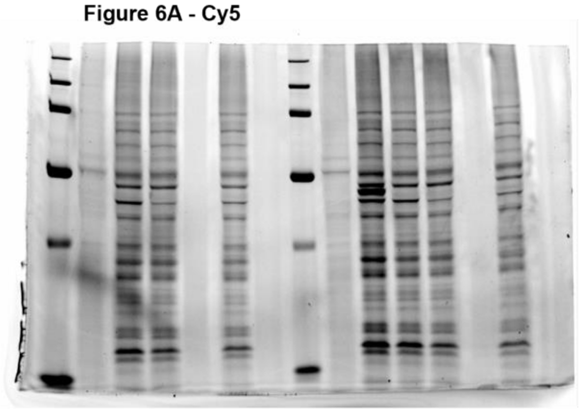

Figure 6C - Cy5

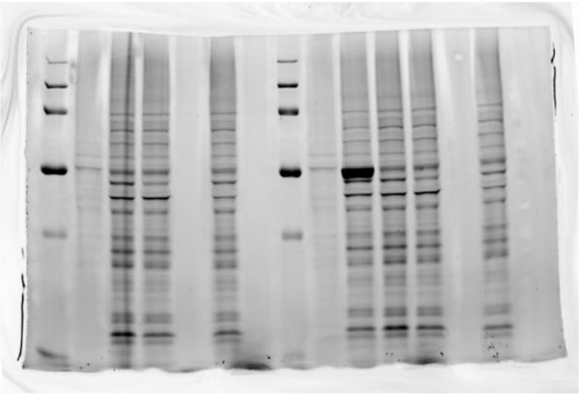

Figure 6A - CM

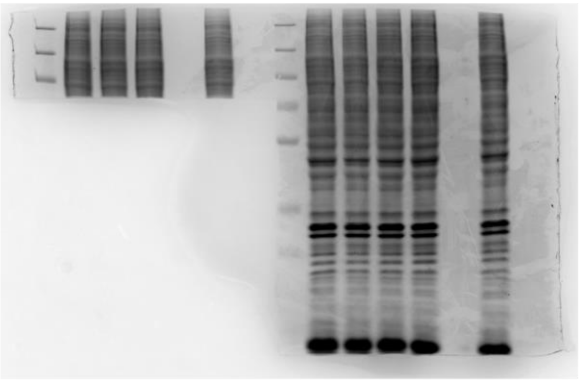

Figure 6C - CM

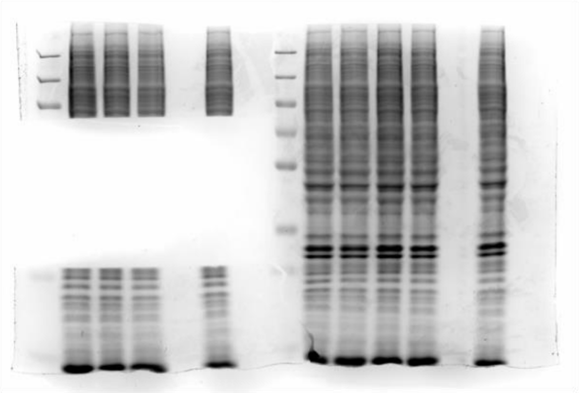

Figure 6C – Chemiluminescence

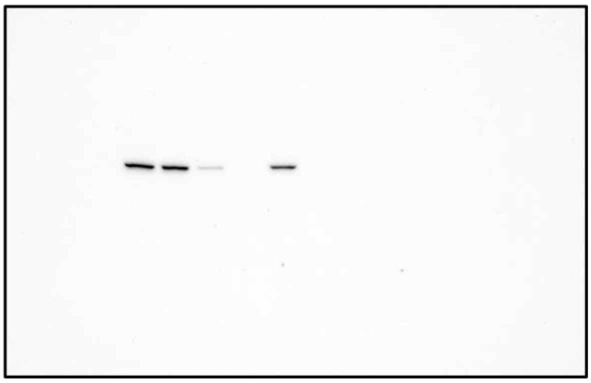

Figure S10. Uncropped gels for Figure 6.

Figure S3B – FP-TAMRA Cy5

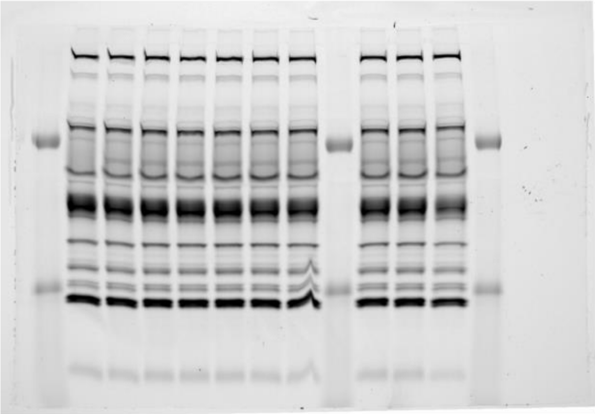

Figure S3B – MB064 Cy5

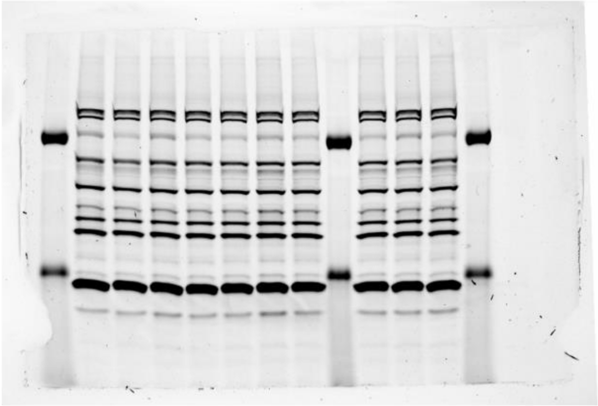

Figure S3B – FP-TAMRA CM

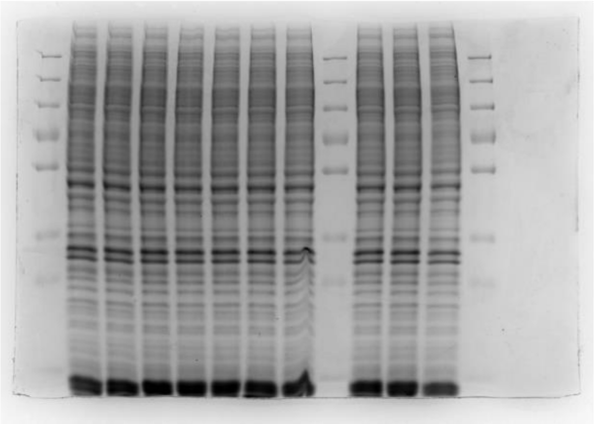

Figure S3B – MB064 CM

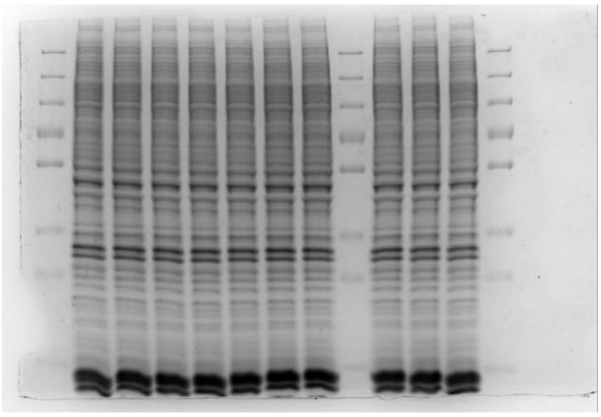

Figure S11. Uncropped gels for Figure S3.

Figure S4B – Cy5

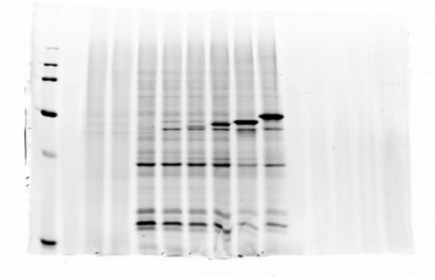

Figure S4B – CM

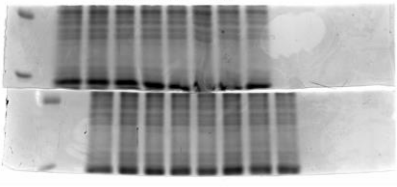

Figure S12. Uncropped gels for Figure S4.

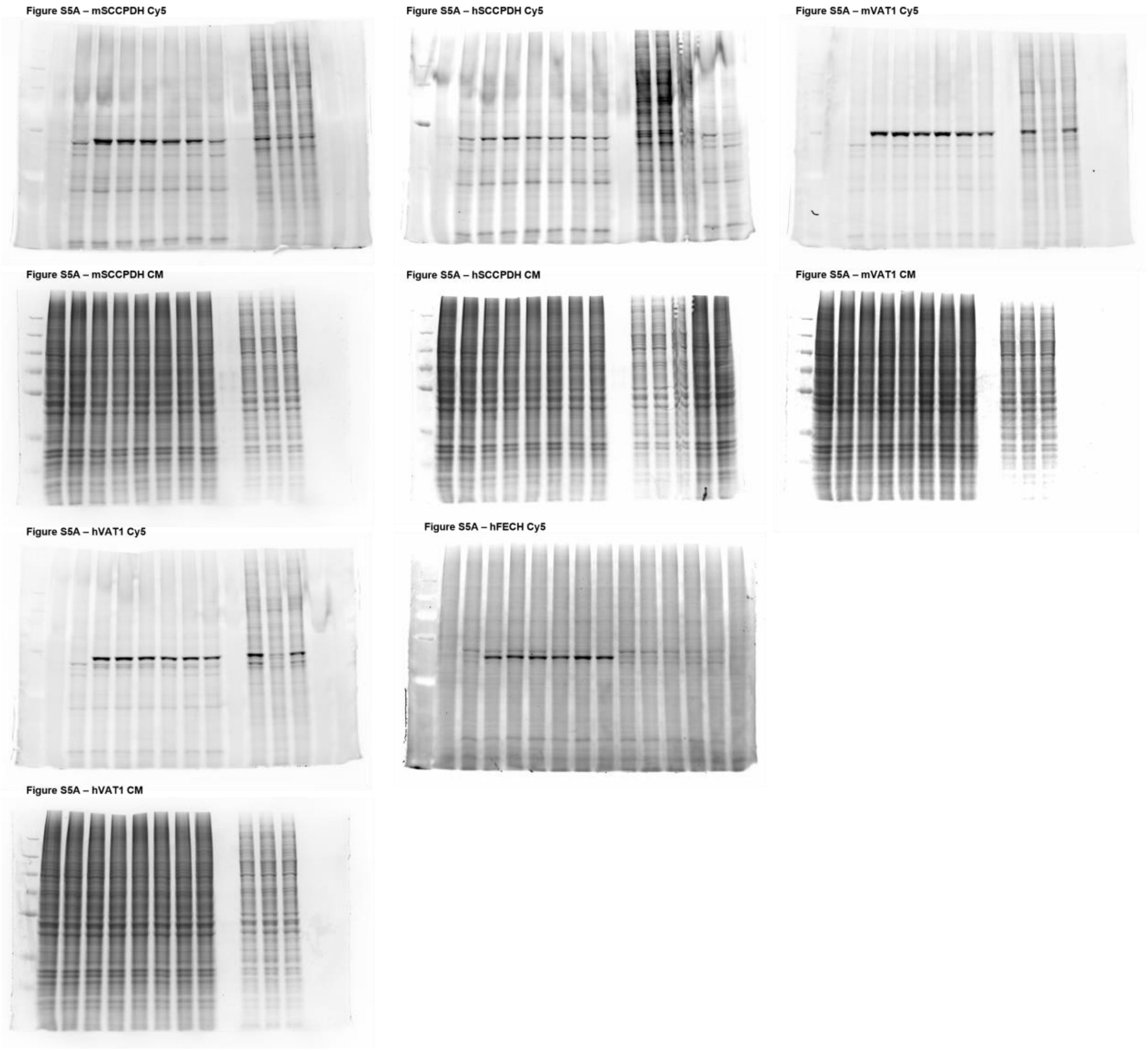

Figure S13. Uncropped gels for Figure S5.

Figure S7A – FP-TAMRA Cy5

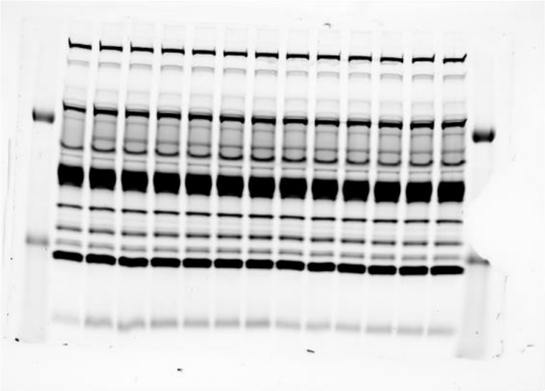

Figure S7B – MB064 Cy5

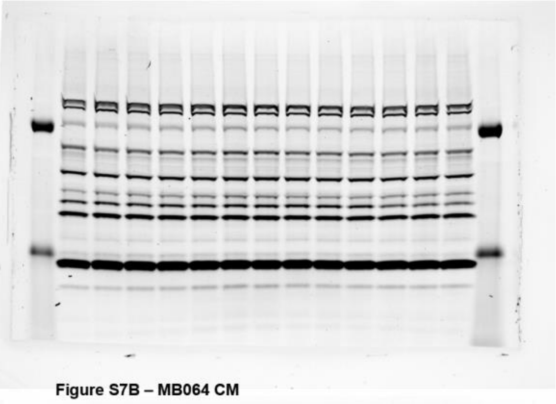

Figure S7A – FP-TAMRA CM

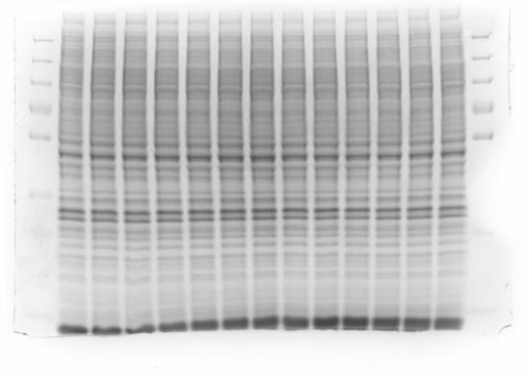

Figure S7B – MB064 CM

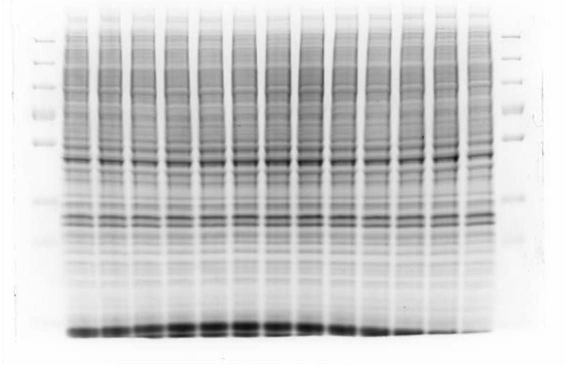

Figure S14. Uncropped gels for Figure S5.

Table S1. Parameters used for Progenesis QI.

Parameter	Value
Lock mass *m*/*z* value	785.8426
Low energy threshold	150 counts
Elevated energy threshold	30 counts
Digest reagent	Trypsin
Missed cleavages	Max 2
Modifications	Fixed carbamidomethyl C, variable oxidation M
FDR less than	1%
Minimum fragments/peptide	2
Minimum fragments/protein	5
Minimum peptides/protein	1
Minimum peptide score for quantification	5.5
Identified ion charges for quantification	$2/3/4/5/6/7^{+}$

Table S2. LC-MS standards and internal standards for lipidomics analysis. The target list includes fatty acids, endocannabinoids, *N*-acylethanolamines (NAEs) and deuterated labeled internal standards. The compound ID is the abbreviation of metabolite name along with number of carbon atoms and number of double bonds in the fatty acid chain of the molecule, respectively. All compounds are analyzed in positive mode except fatty acids in negative mode. Q1 and Q3 are optimized precursor ion and product ion respectively, expressed as m/z. DP and CE are declustering potential (V) and collision energy (V).

**Standards**					
**Abbreviation**	**Metabolite**	**Q1**	**Q3**	**DP, CE**	**Polarity**
**1 & 2-AG (20:4)**	1-Arachidonoyl Glycerol	379.21	287.20	45, 10	+
**2-LG (18:2)**	2-Linoleoyl Glycerol	357.34	247.50	48, 10	+
**2-OG (18:1)**	2-Oleoyl Glycerol	357.34	247.50	40, 12	+
**AEA (20:4)**	Arachidonoyl Ethanolamide	348.40	62.02	35, 16	+
**DEA (22:4)**	Docosatetraenoyl Ethanolamide	376.38	61.92	55, 18	+
**DGLEA (18:3)**	Dihomo-γ-Linolenoyl Ethanolamide	350.38	61.98	40, 14	+
**DHEA (22:6)**	Docosahexaenoyl Ethanolamide	372.38	62.01	50, 14	+
**EPEA (20:5)**	Eicosapentaenoyl Ethanolamide	346.34	61.98	36.16	+
**LEA (18:2)**	Linoleoyl Ethanolamide	324.34	61.98	35, 14	+
**OEA (18:1)**	Oleoyl Ethanolamide	326.4	62.01	45, 16	+
**PDEA (15:0)**	Pentadecanoyl Ethanolamide	286.34	62.01	45, 12	+
**PEA (16:0)**	Palmitoyl Ethanolamide	300.34	61.98	42, 14	+
**POEA (16:1)**	Palmitoleoyl Ethanolamide	298.34	62.01	45, 14	+
**SEA (18:0)**	Stearoyl Ethanolamide	328.38	61.98	45, 16	+
**AA (20:4)**	Arachidonic Acid	302.28	259.30	-40, -12	-
**PA (FA 16:0)**	Palmitic Acid	255.33	237.24	-50, -20	-
**OA (FA 18:1)**	Oleic Acid	281.34	263.31	-50, -20	-
**LA (FA 18:2)**	Linoleic Acid	279.34	261.25	-64, -16	-
**GLA (FA 18:3)**	γ-Linolenic Acid	277.30	58.00	-60, -20	-
**ETA (FA 20:3, (ω−3)**	Eicosatrienoic Acid	305.28	306.09	-60, -18	-
**DGLA (FA 20:3, (ω−6)**	Dihomo-γ-Linolenic Acid (20:3)	305.28	306.03	-66, -18	-
**EPA (FA 20:5,(ω−3)**	Eicosapentaenoic Acid	301.34	257.30	-60, -10	-
**DHA (FA 22:6, (ω−3)**	Docosahexaenoic Acid	327.28	283.31	-60, -10	-
**Internal standards**					
**Abbreviation**	**Metabolite**	**Q1**	**Q3**	**DP, CE**	**Polarity**
**2-AG-d8 (20:4)**	2-Arachidonoyl Glycerol-d8	387.38	294.20	45, 10	+
**AEA-d8 (20:4)**	Arachidonoyl Ethanolamide-d8	356.38	62.79	35, 16	+
**DHEA-d4 (22:6)**	Docosahexaenoyl Ethanolamide-d4	376.38	66.01	50, 14	+
**LEA-d4 (18:2)**	Linoleoyl Ethanolamide-d4	328.34	66.01	35, 16	+
**OEA-d4 (18:1)**	Oleoyl Ethanolamide-d4	330.38	66.01	45, 16	+
**PEA-d5 (16:0)**	Palmitoyl Ethanolamide-d5	305.34	61.98	42, 16	+
**SEA-d3 (18:0)**	Stearoyl Ethanolamide-d3	331.38	61.91	45, 16	+
**EPEA-d4 (20:5)**	Eicosapentaenoyl Ethanolamide-d4	350.34	66.08	36, 18	+
**AA-d8 (20:4)**	Arachidonic Acid-d8	311.34	267.30	-40, -12	-
**PA (16:0)-d31**	Palmitic Acid-d31	286.50	266.37	-40, -22	-

Table S3. sgRNA targets, sgRNA oligos (top, bottom) and T7E1 primers (forward, reverse).

sgRNA Target	Construct	Primer Sequences
***Vat1* – Exon 1**	1162	Top: CACCGTTCGCAGCCCCCGACAGTCG<br>Bottom: AAACCGACTGTCGGGGGCTGCGAAC<br>Forward: TCAGGGTACCTATCAGTCACACGCACGTACAC<br>Reverse: CCATGGGCCCGTAGTCGGTCGTACAGCCCTT
**– Exon 2**	1163	Top: CACCGAGACCGGGCCATAGCGTCT<br>Bottom: AAACAGACGCTATGGCCCGGTCTC<br>Forward: TCAGGGTACCGCTGTGAGGACTGACTGAACAC<br>Reverse: CCATGGGCCCGTGTCTAAATGTTACCACGGCA
***Sccdph* – Exon 1**	1164	Top: CACCGAGGCGCCGAACACCACCAGG<br>Bottom: AAACCCTGGTGGTGTTCGGCGCCTC<br>Forward: TCAGGGTACCGCTTCAGGGGAACCAAGAG<br>Reverse: CCATGGGCCCGCCGTGTTACCCAGTTTCTG
**– Exon 2**	1165	Top: CACCGTTACCGGTCCTACGCAGTTG<br>Bottom: AAACCAACTGCGTAGGACCGGTAAC<br>Forward: TCAGGGTACCCCATCACTGACCACTATAGGCA<br>Reverse: CCATGGGCCCACTGAACAAAACTTGTCGGGTT

Experimental procedures

General

Lipids were purchased from Cayman Chemicals and stored as 10 mM ethanolic stocks under nitrogen at -80 °C, except glycerides, which were dissolved in acetonitrile. Inhibitors were purchased from Cayman Chemicals or Sigma Aldrich and stored as 10 mM DMSO stocks at -20 °C. FP-TAMRA was purchased from Thermo Fisher. MB064[2] and 3-(but-3-yn-1-yl)-3-(2-iodoethyl)-3*H*-diazirine[3] were prepared as previously reported in literature. All other reagents were purchased from Sigma Aldrich or Cayman Chemicals unless otherwise specified.

Cloning

DNA oligos were purchased at Sigma Aldrich or Integrated DNA Technologies. Cloning reagents were from Thermo Fisher. Full-length cDNA encoding human and murine SCCPDH and VAT1, as well as human FECH was obtained from Source Bioscience. Expression constructs were generated by PCR amplification and restriction/ligation cloning into a pcDNA3.1 vector, in frame with a C-terminal FLAG tag. All plasmids were isolated from transformed XL10-Gold or DH10B competent cells (prepared using *E. coli* transformation buffer set, Zymo Research) using plasmid isolation kits following the supplier's protocol (Qiagen). All sequences were verified by Sanger sequencing (Macrogen).

Viability assay

$2.0x10^5$ Neuro-2a cells were seeded in 500 µL medium on a 24-well plate 24 h prior. Then, 55 µL PBS supplemented with 5 mg/mL 3-(4,5-dimethylthiazol-2-yl)-2,5-diphenyltetrazolium bromide was added for 0.5 mg/mL final and the plate was incubated at 37 °C for 3 h. The medium was aspirated and the formed formazan crystals were dissolved in 200 µL DMSO by shaking the plates at 800 rpm for 5 min. Absorbance was measured at 450 nm in a CLARIOstar plate reader (BMG Labtech) and data was analyzed using Graphpad Prism 8.1.1.

Activity-based protein profiling

To perform comparative ABPP, 39 µL of lysate (39 µg protein) was mixed with 1 µL of indicated compound (40X stock in DMSO) before probe addition. Final concentration of probes were 500 nM for FP-TAMRA and 2 µM for MB064. The probes were incubated for 30 min at rt before quenching the reaction with 4X Laemmli buffer for 30 min at rt. Labelled proteins were resolved by SDS-PAGE (10% acrylamide gel, ±80 min, 180 V) along with protein marker (PageRuler™ Plus, Thermo Fisher). In-gel fluorescence was measured in the Cy3- and Cy5-channel (Chemidoc™ MP, Bio-Rad) and the gels were subsequently stained with Coomassie and imaged as a loading control for normalization of fluorescence intensity. Band intensities were quantified using Image Lab 6.0.1 (BioRad).

CRISPR/Cas9 KO generation

Guide design & constructs

Two sgRNAs, in early exons of the *Vat1* and *Sccpdh* genes, with high efficiency and specificity as predicted by CHOPCHOP v2 online web tool were selected.[4] Guides were cloned into the *Bbs*I restriction site of plasmid px330-U6-Chimeric_BB-CBh-hSpCas9 (a kind gift from Feng Zhang, Addgene plasmid #42230) as previously described.[5,6] Primers are annotated in Table S3.

CRISPR/Cas9-mediated knockout population generation

Note: Neuro-2a cells display high level of heterogeneity upon clonal isolation.[7] To circumvent this issue, sequential transfections were used to generate a high efficiency knockout cell population.

Neuro-2a cells were transfected sequentially (3 times within the course of 10 days) to yield populations with a high knockout efficiency. Cells were seeded at day 1, 4, and 7 and transfected at day 2, 5, and 8. Samples for T7E1 assays were harvested at day 4, 7, and 10 and after several weeks of culturing the cells. One day prior to the first transfection, Neuro-2a cells were seeded to a 6-well plate to reach 80% confluence at the time of transfection. Prior to transfection, culture medium was aspirated and 2 mL of fresh medium was added. A 5:1 (m/m) mixture of PEI (17.5 µg per well) and plasmid DNA (total 3.5 µg per well) was prepared in serum-free culture medium (250 µL each) and incubated (15 min, rt). Transfection was performed by dropwise addition of the PEI/DNA mixture to the cells. 24 h post-transfection the culture medium was refreshed. 48 h post-transfection a small amount of cells was harvested for analysis by T7E1 assay and ABPP, while the remainder was kept in culture under standard conditions for following transfections. After three transfection rounds, the cells were cultured according to standard protocol. Ampoules of knockdown populations were prepared (complete DMEM, 10% DMSO) and stored at -150 °C. Efficiency of knockdown was checked over time. Cells were discarded after 3 months of culture.

T7E1 assay

Genomic DNA was obtained by mixing 50 µL QuickExtract™ (Epicentre) with cell pellet (~10% of a well from a 6-well plate). The samples were incubated at 65 °C for 6 min, mixed by vortexing and incubated at 98 °C for 2 min. Genomic DNA extracts were diluted in sterile water and directly used in PCR reactions. Genomic PCR reactions were performed on 5 µL isolated genomic DNA extract using Phusion High-Fidelity DNA Polymerase (Thermo Fisher) in Phusion GC buffer Green (Thermo Fisher) in a final volume of 45 µL, primers are annotated in Table S3.

For the T7E1 assay, genomic PCR products (20 µL) were denatured and reannealed in a thermocycler using the following program: 5 min at 95 °C, 95 to 85 °C using a ramp rate of -2 °C/s, 85 °C to 25 °C using a ramp rate of -0.2 °C/s. Annealed PCR product (8.5 µL) was mixed with NEB2 buffer (1 µL) and T7 endonuclease I (5 U, 0.5 µL; New England Biolabs), followed by a 30 min incubation at 37 °C. Digested PCR products were analyzed using agarose gel electrophoresis with ethidium bromide staining. A sample without T7 endonuclease I was taken along as control. Agarose gels were analyzed using Image Lab 6.0.1 (BioRad).

NAPE-PLD surrogate substrate-based fluorescence assay

The NAPE-PLD activity assay was performed according to a previously reported method with minor adjustments.[1,8] Purified recombinant MBP-tagged (N-terminal) and His_6-tagged (C-terminal) human Δ47-NAPE-PLD from *Escherichia coli*, a kind gift from Dr. Piomelli, was diluted to 25 nM in assay buffer (50 mM Tris pH 7.5, 150 mM NaCl, 0.02% Triton X-100).[9] The substrate PED6 (Invitrogen, D23739, 1 mM stock in DMSO) was consecutively diluted in DMSO (5X) and in assay buffer (10X), to make a 20 µM working solution. Inhibitor solutions (50X) were prepared in DMSO. The assay was performed in a dark 96-well plate (flat bottom, Greiner), in a final volume of 50 µL. Inhibitor or DMSO was incubated with enzyme (2.5 nM final) for 30 min at 37 °C. Then, PED6 was added (2 µM final) and the measurement was started immediately on a CLARIOstar plate reader (BMG Labtech) at 37 °C (excitation

474-490 nm, emission 510-550 nm), scanning every 2 min for 1 hour. Negative control wells containing no enzyme were used for background subtraction. The measurements were performed in n = 4.

Statistical analysis

Unless otherwise noted, all replicates represent biological replicates and all data represent means ± SEM. Statistical significance was determined using Student's t-tests (two-tailed, unpaired) or one-way ANOVA with Dunnett multiple comparisons correction. *** $p < 0.001$; ** $p < 0.01$; * $p < 0.05$; n.s. if $p > 0.05$. All statistical analysis were conducted using Graphpad Prism 8.1.1 or Microsoft Excel.

Synthesis

General remarks

Dry solvents were prepared by storage on activated 4 Å molecular sieves for at least 24 hours. The reactions were performed under an inert atmosphere of nitrogen gas unless stated otherwise. All reagents were purchased from Alfa Aesar, Sigma Aldrich/Merck or Acros and used without further purification. Flash column chromatography was performed using SiliCycle silica gel type SiliaFlash P60 (230-400 mesh). TLC analysis was performed on Merck silica gel 60/Kieselguhr F254, 0.25 mm. Compounds were visualized using $KMnO_4$ stain (K_2CO_3 (40 g), $KMnO_4$ (6 g), and water (600 mL)).

^1H NMR and ^{13}C NMR spectra were recorded on a Bruker AV-400 (400 MHz) or AV-500 (500 MHz) spectrometer. Chemical shift values are reported in ppm relative to the tetramethylsilane signal for ^1H NMR (δ = 0 ppm) and relative to the solvent signal of $CDCl_3$ for ^{13}C NMR (δ = 77.16 ppm). Data are reported as follows: Chemical shifts (δ), multiplicity (s = singlet, d = doublet, dd = doublet of doublets, ddt = doublet of doublet of triplets, td = triplet of doublets, t = triplet, q = quartet, p = pentet, bs = broad singlet, m = multiplet), coupling constants *J* (Hz), and integration. High resolution mass spectra (HRMS) were recorded by direct injection on a q-TOF mass spectrometer (Synapt G2-SI) equipped with an electrospray ion source in positive mode with Leu-enkephalin (m/z = 556.2771) as an internal lock mass. The instrument was calibrated prior to measurement using the MS/MS spectrum of [Glu^1]-fibrinopeptide B.

Ethyl (2*E*,4*E*)-dodeca-2,4-dienoate (5)

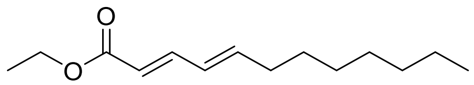

To a cooled (0 °C) suspension of sodium hydride (0.518 g, 12.94 mmol) in dry THF (30 mL) was added a solution of ethyl 2-(diethoxyphosphoryl)acetate (2.90 g, 12.94 mmol in dry THF (10 mL)) dropwise and the reaction was stirred for 10 min. It was then cooled to -78 °C and a solution of (*E*)-dec-2-enal (1.98 mL, 10.8 mmol) in dry THF (10 mL) was added dropwise and the reaction was allowed to reach rt overnight. The reaction was quenched with water (150 mL) and the mixture was extracted with EtOAc (2 x 150 mL). The combined organic layers were washed with brine (100 mL), dried over $MgSO_4$, filtered and concentrated under reduced pressure. Purification of the residue by column chromatography (pentane/Et_2O = pentane to 9:1) afforded the title compound as a clear oil (1.5281 g, 6.81 mmol, 63%). R_f = 0.4 (pentane/Et_2O = 19:1); ^1H NMR (400 MHz, $CDCl_3$) δ 7.35 – 7.19 (m, 1H), 6.23 – 6.06 (m, 2H), 5.78 (d, *J* = 15.4 Hz, 1H), 4.20 (q, *J* = 7.1 Hz, 2H), 2.16 (q, *J* = 7.1 Hz, 2H), 1.48 – 1.38 (m, 2H), 1.38 – 1.19 (m, 11H), 0.88 (t, *J* = 6.7 Hz, 3H); ^{13}C NMR (101 MHz, $CDCl_3$) δ 167.48, 145.27, 144.96, 128.44, 119.25, 60.30, 33.14, 31.91, 29.28, 29.24, 28.85, 22.78, 14.46, 14.23. Spectra were consistent with previously reported data.[10]

(2*E*,4*E*)-dodeca-2,4-dienoic acid (6)

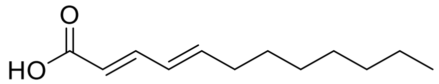

To a solution of ester **5** (1.514 g, 6.75 mmol) in MeOH (17 mL) was added 2 M aq. NaOH (6.75 mL, 13.50 mmol) and the reaction was stirred at 60 °C for 30 min. Solvent was removed under reduced pressure and the reaction was diluted with water (10 mL), washed with Et_2O (20 mL), acidified with 1 M aq. HCl to pH <4 and extracted with EtOAc (2 x 20 mL). The combined organic layers were washed with brine (20 mL), dried over $MgSO_4$, filtered and concentrated under reduced pressure to afford the title compound as an off-white solid (1.324 g, 6.75 mmol, quant.). R_f = 0.6 (pentane/EtOAc = 1:1 + 0.5% AcOH); ^1H NMR (400 MHz, $CDCl_3$) δ 7.40 – 7.30 (m, 1H), 6.23 – 6.16 (m, 2H), 5.78 (d, *J* = 15.3 Hz, 1H), 2.23 – 2.13 (m, 2H), 1.48 – 1.39 (m, 2H), 1.37 – 1.19 (m, 8H), 0.87 (t, *J* = 6.7 Hz, 3H); ^{13}C NMR (101 MHz, $CDCl_3$) δ 172.81, 147.77, 146.56, 128.33, 118.27, 33.22, 31.91, 29.29, 29.24, 28.76, 22.78, 14.24. Spectra were consistent with previously reported data.[10]

(2*E*,4*E*)-*N*-(3,4-dimethoxyphenethyl)dodeca-2,4-dienamide (WOBE437, 1)

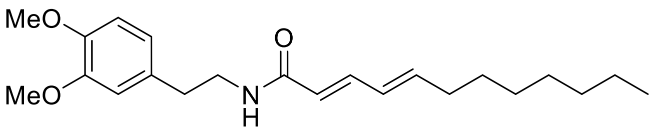

To a solution of carboxylic acid **6** (0.2500 g, 1.274 mmol) and 2-(3,4-dimethoxyphenyl)ethan-1-amine (0.254 g, 1.401 mmol) in DCM (2.5 mL) was added HOAt (0.087 g, 0.637 mmol) and EDC (0.293 g, 1.528 mmol) and the reaction was stirred for 45 min. Then, it was diluted with DCM (100 mL) and sat. aq. $NaHCO_3$ (100 mL). The layers were separated and the aq. layer was extracted with DCM (2 x 100 mL). The combined organic layers were dried over $MgSO_4$, filtered and concentrated under reduced pressure. Purification of the residue by column chromatography (pentane/EtOAc = 1:4 to 1:1) afforded the title compound (WOBE437, **1**) as a white solid (0.3585 g, 0.997 mmol, 78%). R_f = 0.5 (pentane/EtOAc = 1:1 + 0.5% AcOH); ^{1}H NMR (400 MHz, DMSO-d_6) δ 8.00 (t, J = 5.7 Hz, 1H), 6.97 (dd, J = 15.1, 10.6 Hz, 1H), 6.85 (d, J = 8.2 Hz, 1H), 6.79 (d, J = 2.0 Hz, 1H), 6.70 (dd, J = 8.2, 2.0 Hz, 1H), 6.17 (dd, J = 15.2, 10.7 Hz, 1H), 6.06 (dt, J = 15.1, 6.7 Hz, 1H), 5.89 (d, J = 15.1 Hz, 1H), 3.72 (s, 3H), 3.70 (s, 3H), 3.33 – 3.28 (m, 2H), 2.66 (t, J = 7.3 Hz, 2H), 2.11 (q, J = 7.1 Hz, 2H), 1.43 – 1.32 (m, 2H), 1.32 – 1.17 (m, 8H), 0.89 – 0.82 (t, J = 6.7 Hz, 3H); ^{13}C NMR (101 MHz, DMSO) δ 165.21, 148.57, 147.20, 141.69, 139.12, 131.93, 128.60, 123.29, 120.42, 112.47, 111.83, 55.49, 55.34, 40.42, 34.72, 32.26, 31.25, 28.59, 28.53, 28.38, 22.10, 13.97. Spectra were consistent with previously reported data.[10]

(2*E*,4*E*)-*N*-(3-hydroxy-4-methoxyphenethyl)dodeca-2,4-dienamide (7)

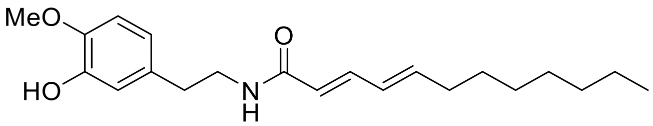

To a cooled (0 °C) solution of 5-(2-aminoethyl)-2-methoxyphenol hydrochloride (0.0836 g, 0.408 mmol) in dry DMF (1.5 mL) was added carboxylic acid **6** (0.088 g, 0.449 mmol), EDC (0.094 g, 0.490 mmol), HOBt (0.066 g, 0.490 mmol) and Et_3N (0.114 mL, 0.817 mmol) after which the reaction was allowed to reach rt. After 3 hours, the reaction was diluted with water (25 mL) and the mixture was extracted with Et_2O (2 x 30 mL). The combined organic layers were washed with brine (4 x 10 mL), dried over Na_2SO_4, filtered and concentrated under reduced pressure. Purification of the residue by column chromatography (pentane/EtOAc/$CHCl_3$ = 4/1/2 + 0.5% AcOH to 2/1/1 + 0.5% AcOH) afforded the title compound as an off-white solid (0.0611 g, 0.177 mmol, 43%). R_f = 0.5 (pentane/EtOAc/$CHCl_3$ = 2/2/1 + 0.5% AcOH); ^{1}H NMR (400 MHz, $CDCl_3$) δ 7.19 (dd, J = 15.0, 10.0 Hz, 1H), 6.82 – 6.75 (m, 2H), 6.67 (dd, J = 8.1, 2.2 Hz, 1H), 6.12 – 6.03 (m, 2H), 5.68 (d, J = 15.0 Hz, 1H), 5.56 (bs, 1H), 3.87 (s, 3H), 3.56 (q, J = 6.6 Hz, 2H), 2.75 (t, J = 6.8 Hz, 2H), 2.17 – 2.12 (m, 2H), 1.47 – 1.35 (m, 2H), 1.35 – 1.21 (m, 8H), 0.88 (t, J = 6.4 Hz, 3H); ^{13}C NMR (101 MHz, $CDCl_3$) δ 166.72, 145.82, 145.43, 143.74, 141.83, 132.19, 128.26, 121.47, 120.32, 115.01, 110.99, 56.12, 40.85, 35.09, 33.10, 31.90, 29.26, 29.23, 28.90, 22.77, 14.22. Spectra were consistent with previously reported data.[10]

(2*E*,4*E*)-*N*-(3-(2-(3-(but-3-yn-1-yl)-3H-diazirin-3-yl)ethoxy)-4-methoxyphenethyl)dodeca-2,4-dienamide (pac-WOBE, 3)

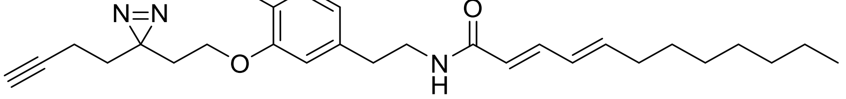

A solution of phenol **7** (16.8 mg, 0.049 mmol), 3-(but-3-yn-1-yl)-3-(2-iodoethyl)-3*H*-diazirine **8** (19.3 mg, 0.078 mmol) and K_2CO_3 (13.4 mg, 0.097 mmol) in dry DMF (0.5 mL) was purged with N_2 and stirred at 60 °C overnight. The mixture was quenched with water (4 mL), extracted with Et_2O (3 x 5 mL), dried over Na_2SO_4, filtered and concentrated under reduced pressure. Phenol **7** and the title compound were separated by column chromatography (MeOH/DCM = 1:1,000 to 1:100) and the recovered phenol **7** was dissolved in dry DMF (0.5 mL) with 3-(but-3-yn-1-yl)-3-(2-iodoethyl)-3*H*-diazirine **8** (19.3 mg, 0.078 mmol) and K_2CO_3 (13.4 mg, 0.097 mmol), purged with N_2 and stirred at 60 °C overnight. The mixture was quenched with water (4 mL), extracted with Et_2O (3 x 5 mL), dried over Na_2SO_4 and concentrated under reduced pressure. It was combined with previously isolated title compound and purified with

column chromatography twice (pentane/EtOAc = 9:1 to 2:1 followed by MeOH/DCM = 1:1,000 to 1:100) to afford the title compound (pac-WOBE, **3**) as a white solid (0.0065 g, 0.014 mmol, 29%). R_f = 0.7 (pentane/EtOAc = 1:1 + 0.5% AcOH); ^{1}H NMR (500 MHz, $CDCl_3$) δ 7.18 (dd, *J* = 15.0, 9.7 Hz, 1H), 6.82 (d, *J* = 8.2 Hz, 1H), 6.76 (dd, *J* = 8.1, 2.0 Hz, 1H), 6.71 (d, *J* = 2.0 Hz, 1H), 6.17 – 6.02 (m, 2H), 5.68 (d, *J* = 15.0 Hz, 1H), 5.43 (bs, 1H), 3.89 – 3.84 (m, 5H), 3.57 (q, *J* = 6.7 Hz, 2H), 2.78 (t, *J* = 6.9 Hz, 2H), 2.14 (q, *J* = 6.9 Hz, 2H), 2.09 (td, *J* = 7.6, 2.6 Hz, 2H), 1.98 (t, *J* = 2.7 Hz, 1H), 1.89 (t, *J* = 6.4 Hz, 2H), 1.76 (t, *J* = 7.6 Hz, 2H), 1.44 – 1.37 (m, 2H), 1.28 (m, 8H), 0.88 (t, *J* = 6.9 Hz, 3H); ^{13}C NMR (126 MHz, $CDCl_3$) δ 166.45, 148.51, 148.22, 143.63, 141.67, 131.67, 128.30, 121.70, 121.63, 114.55, 112.36, 83.06, 69.20, 63.96, 56.26, 40.90, 35.31, 33.28, 33.11, 32.85, 31.92, 29.29, 29.25, 28.95, 26.86, 22.78, 14.23, 13.45. HRMS: Calculated for $[C_{28}H_{39}N_3O_3+H]^+$ 466.3064, found 466.3073.

1H, 13C NMR and HRMS spectra

Ethyl (2*E*,4*E*)-dodeca-2,4-dienoate (5)

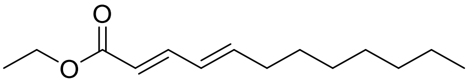

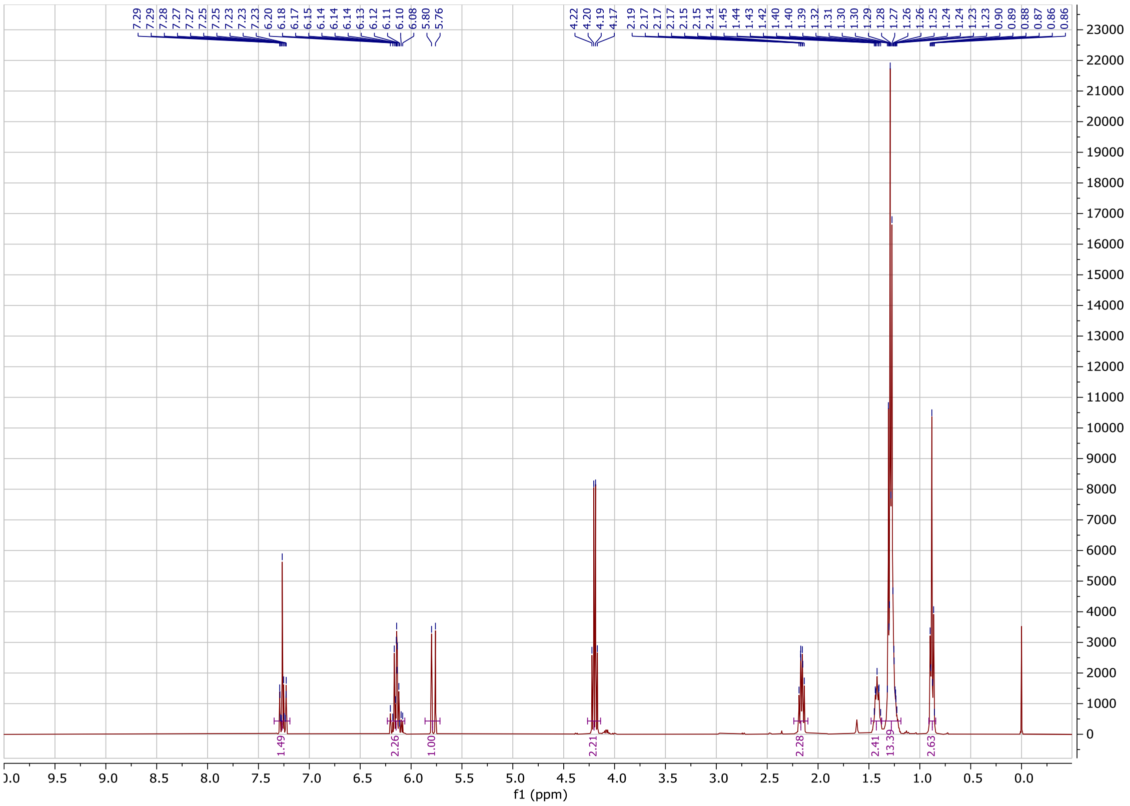

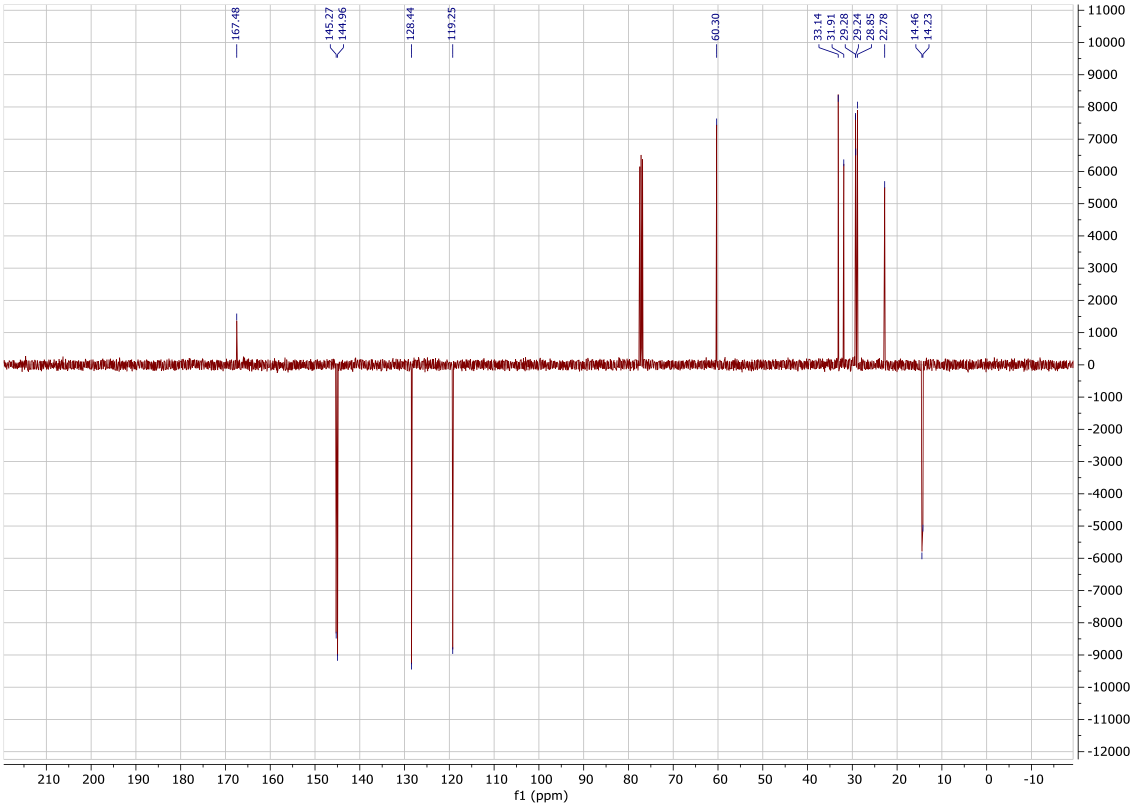

(2*E*,4*E*)-dodeca-2,4-dienoic acid (6)

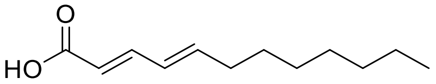

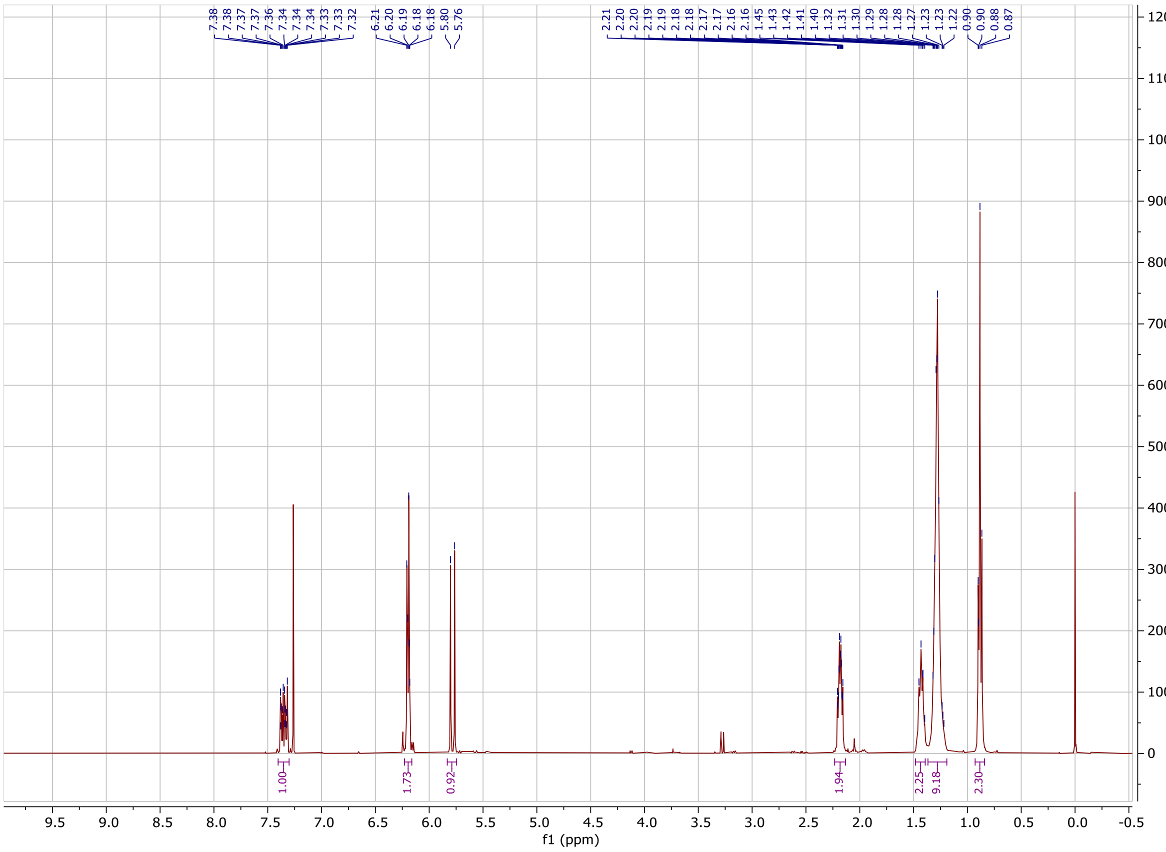

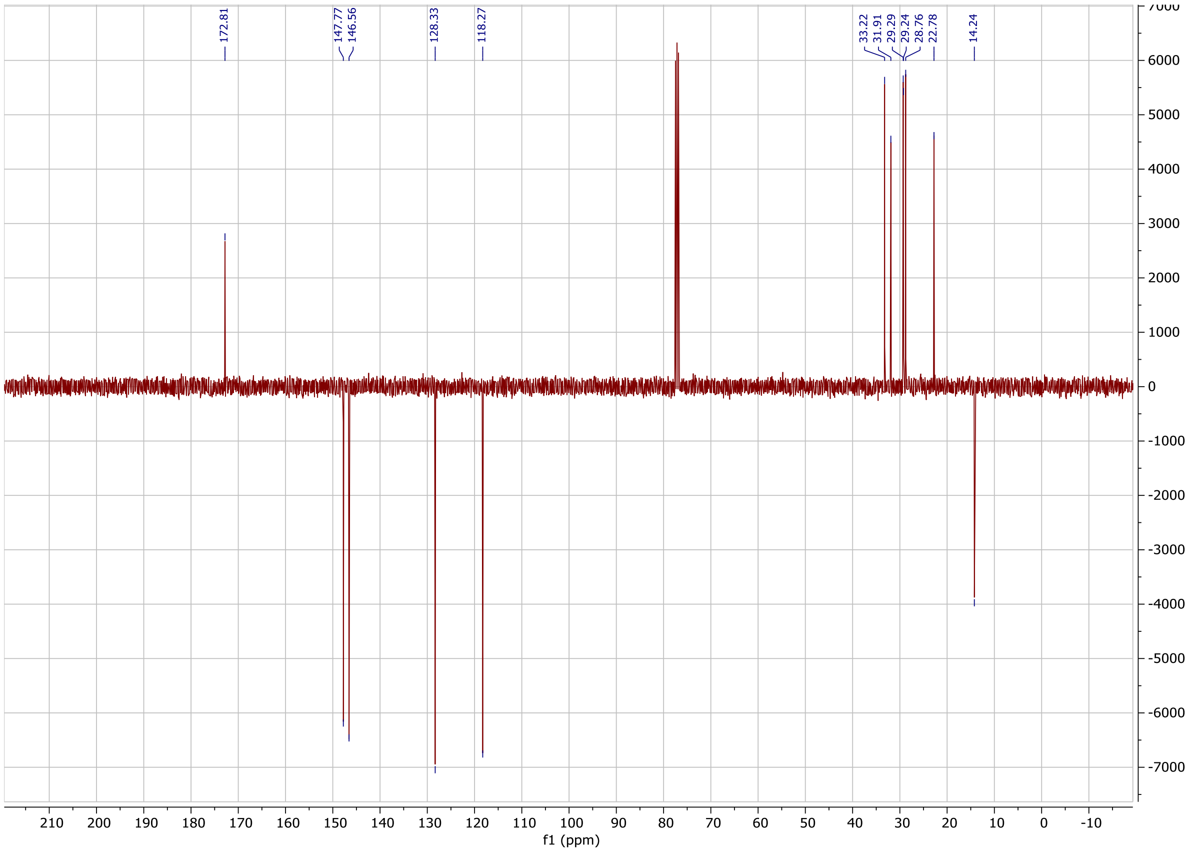

(2*E*,4*E*)-*N*-(3,4-dimethoxyphenethyl)dodeca-2,4-dienamide (WOBE437, 1)

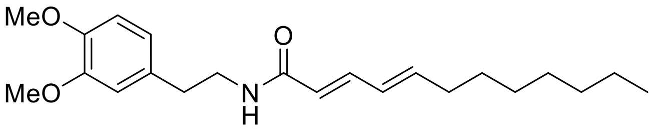

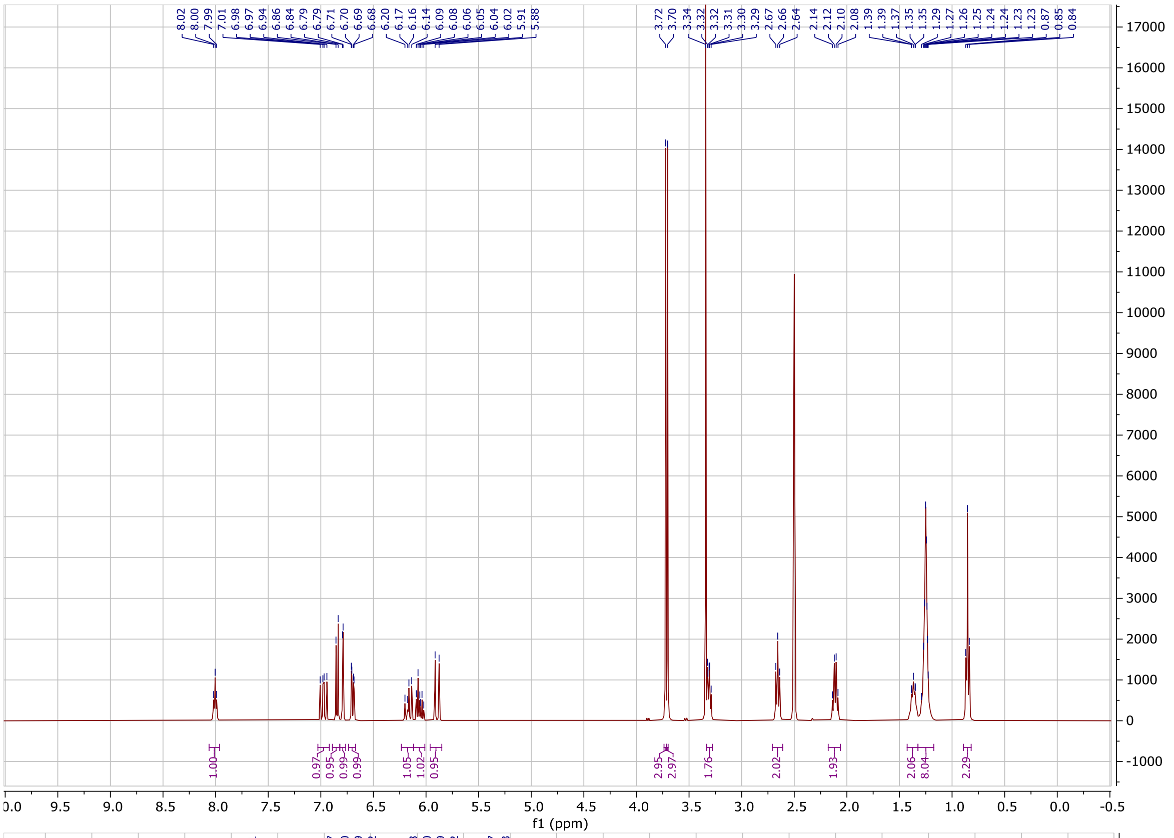

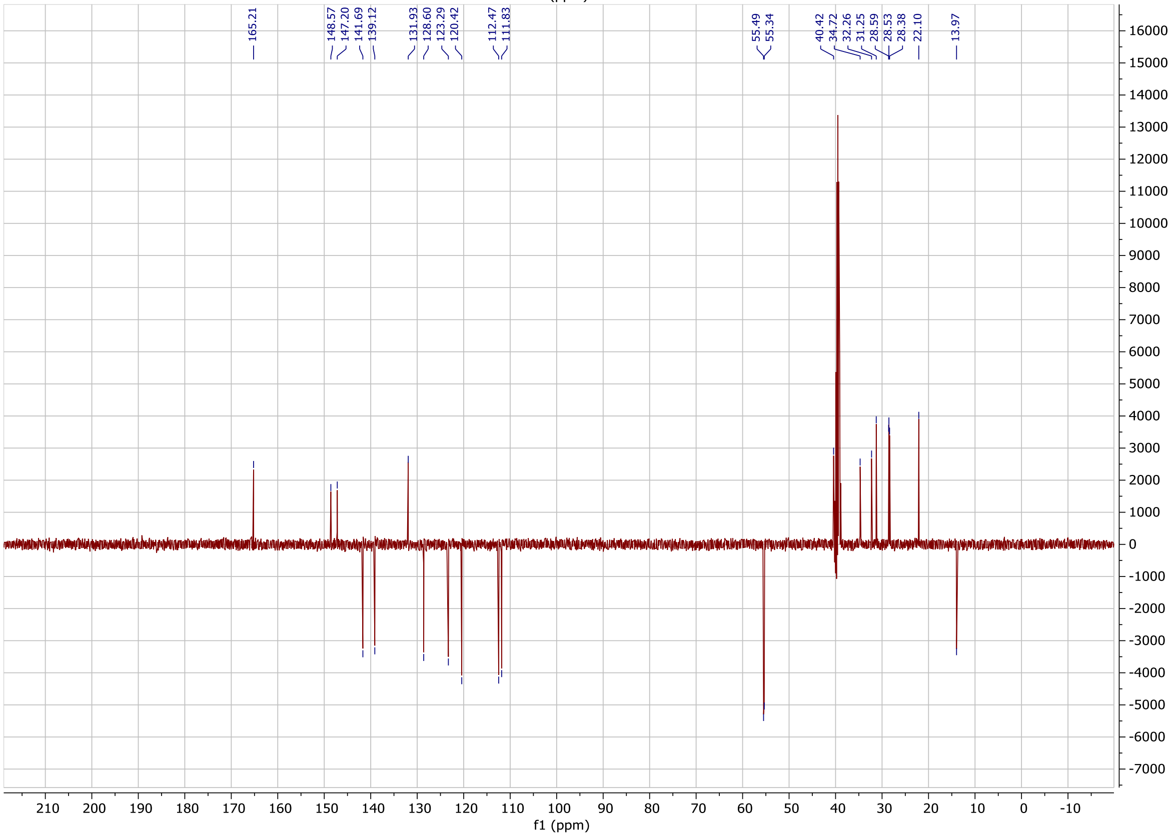

(2*E*,4*E*)-*N*-(3-hydroxy-4-methoxyphenethyl)dodeca-2,4-dienamide (7)

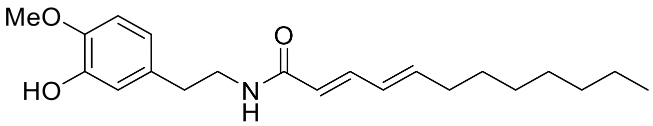

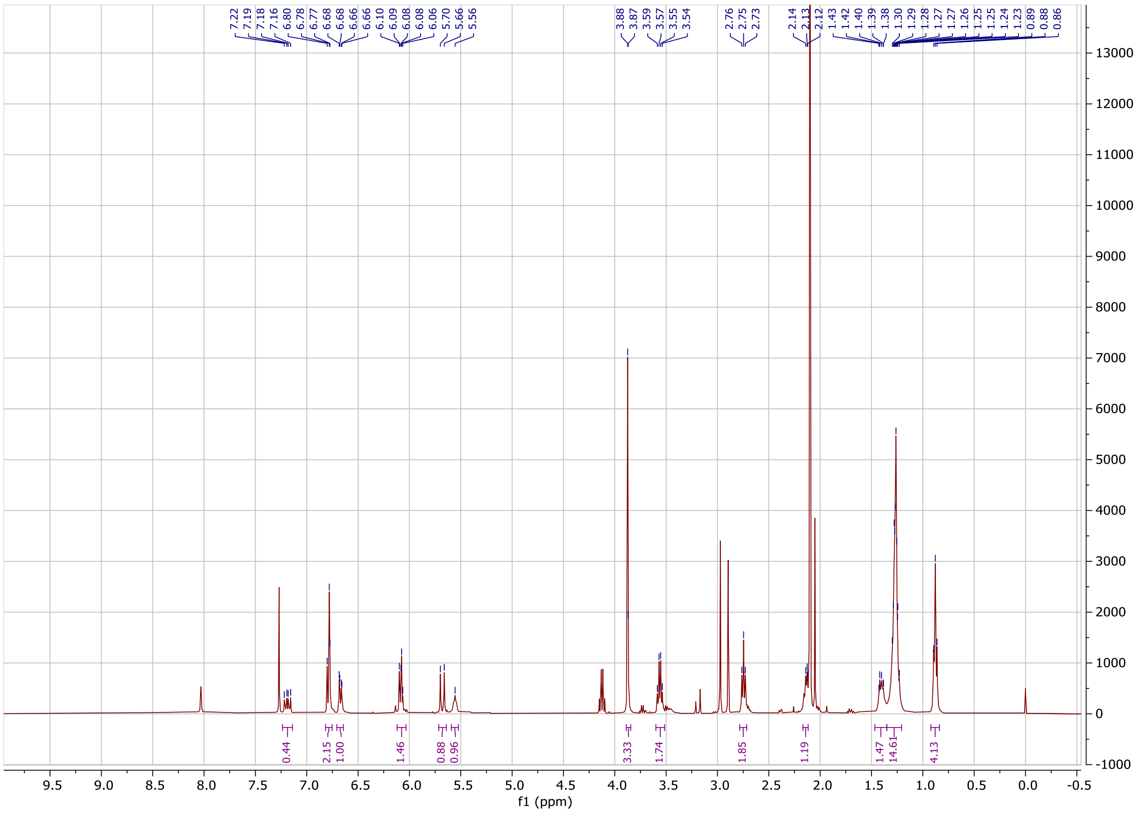

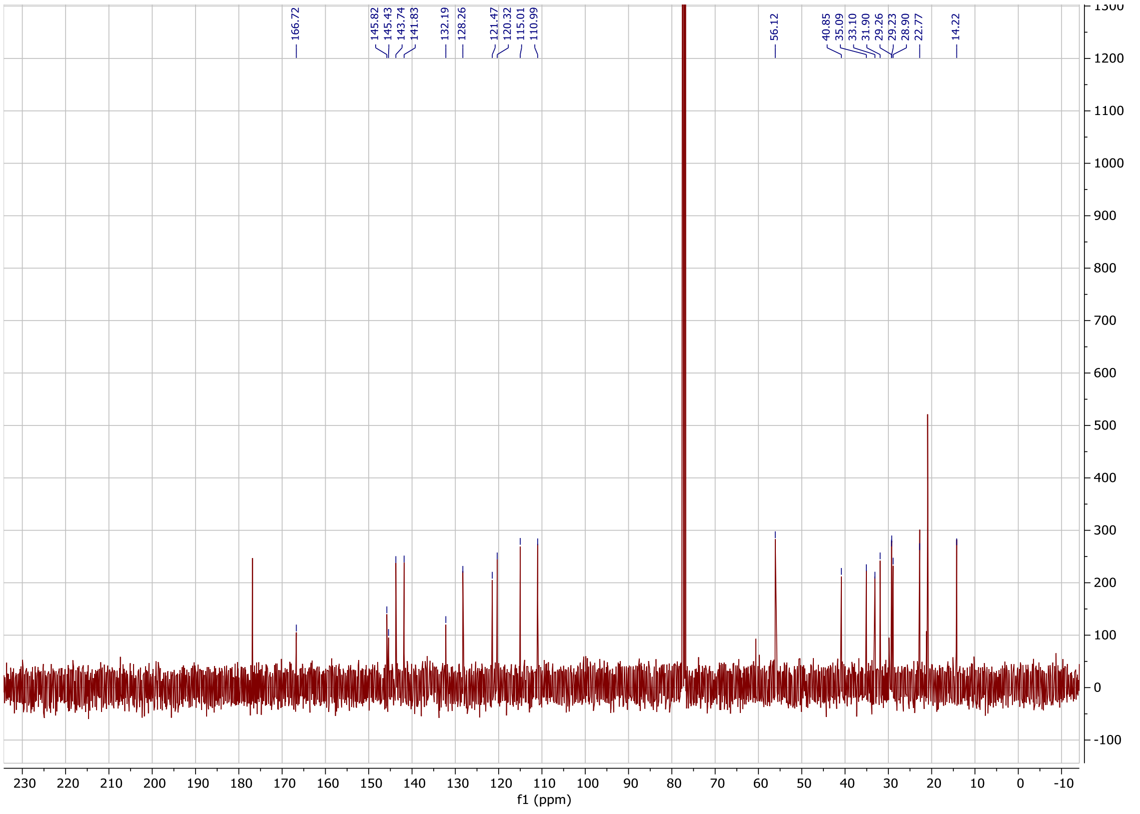

(2*E*,4*E*)-*N*-(3-(2-(3-(but-3-yn-1-yl)-3H-diazirin-3-yl)ethoxy)-4-methoxyphenethyl)dodeca-2,4-dienamide (pac-WOBE, 3)

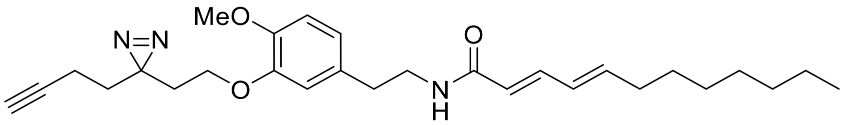

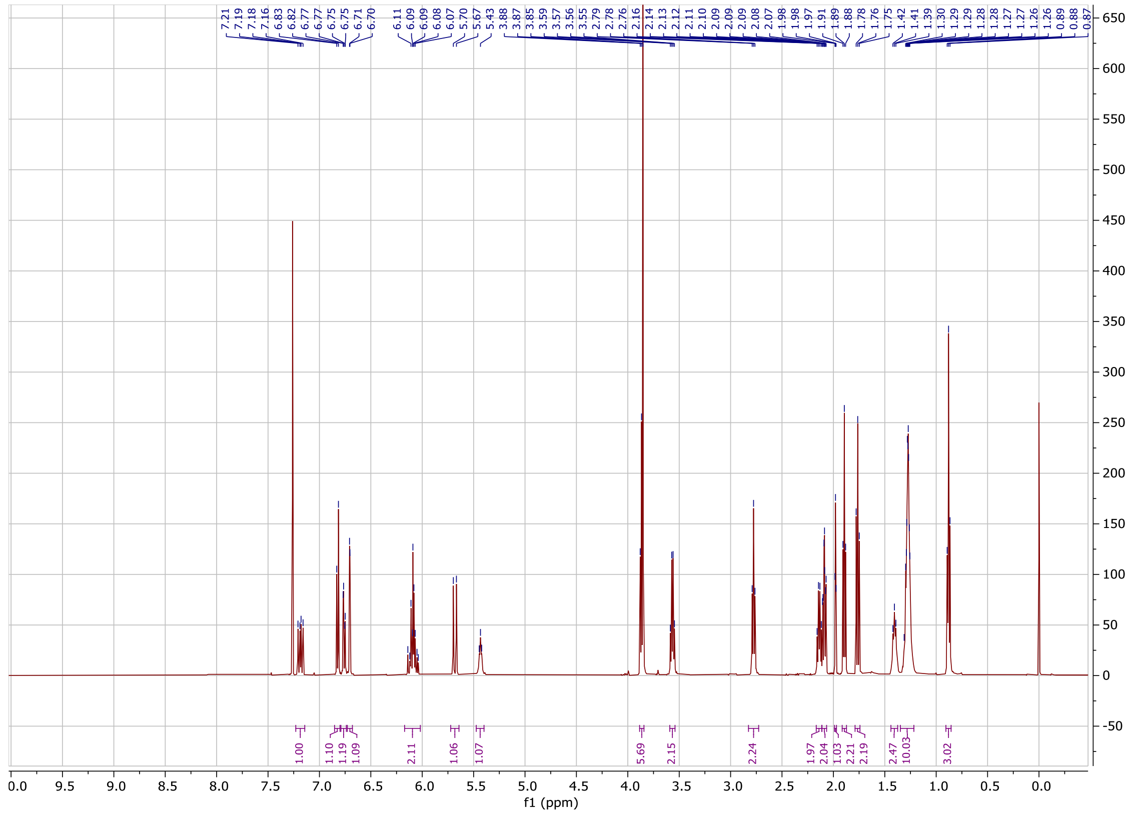

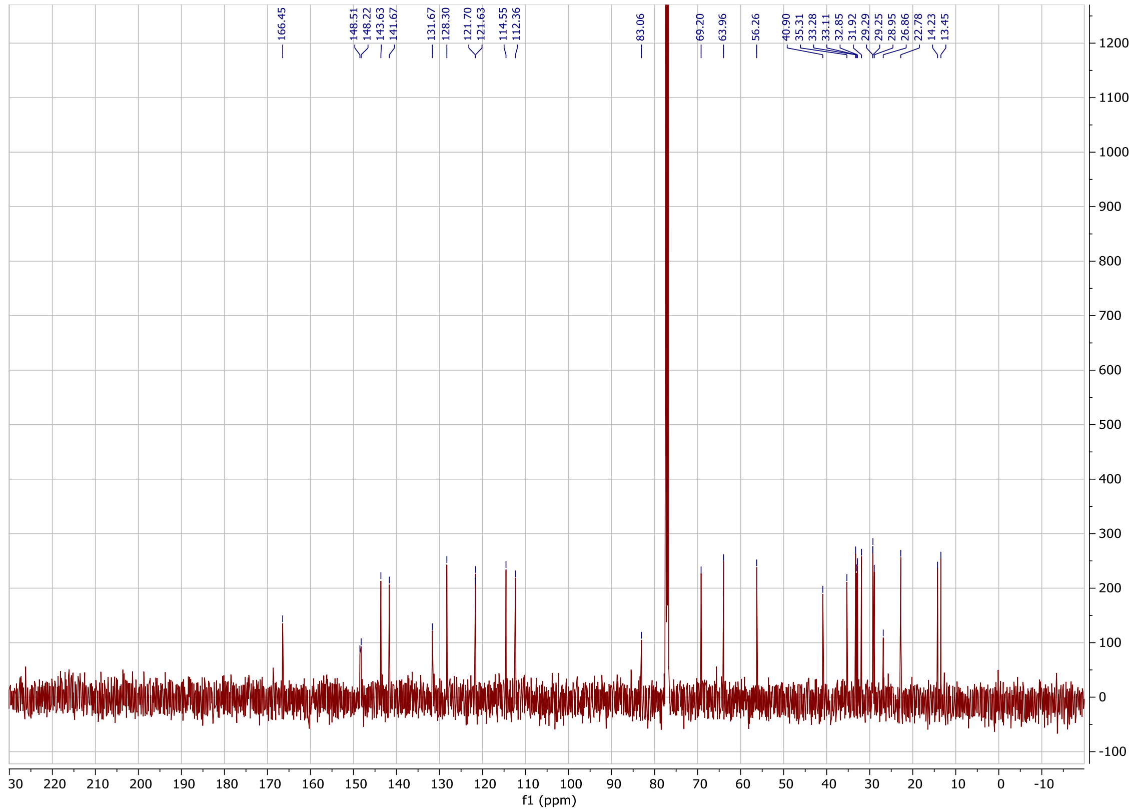

(2*E*,4*E*)-*N*-(3-(2-(3-(but-3-yn-1-yl)-3H-diazirin-3-yl)ethoxy)-4-methoxyphenethyl)dodeca-2,4-dienamide (pac-WOBE, 3) HRMS spectrum

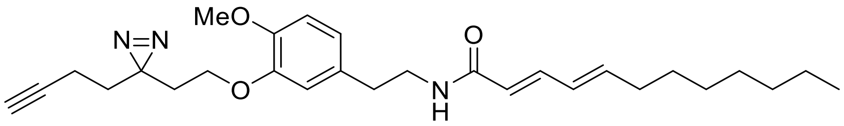

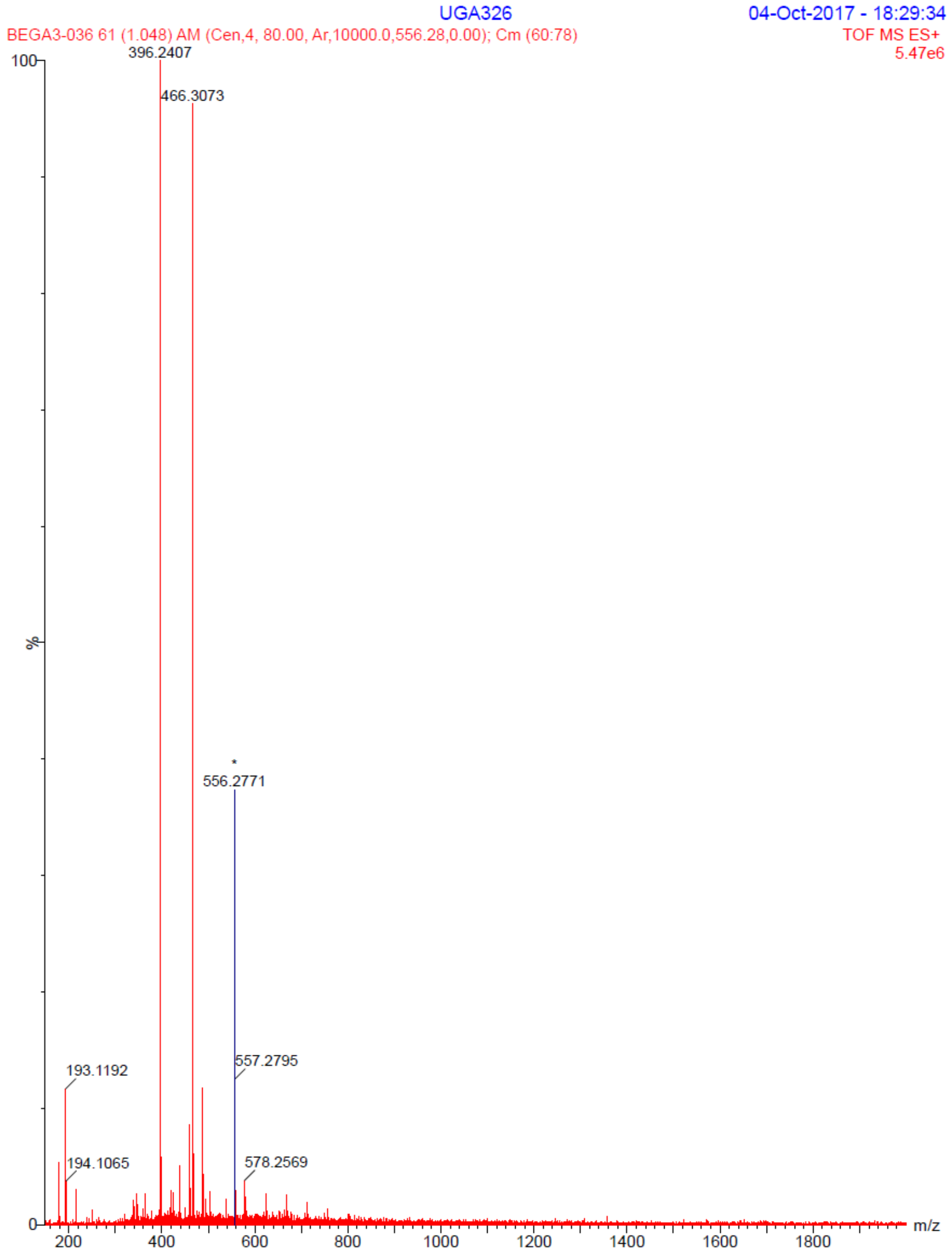

References

(1) E. D. Mock, M. Mustafa, O. Gunduz-Cinar, R. Cinar, G. N. Petrie, V. Kantae, X. Di, D. Ogasawara, Z. V. Varga, J. Paloczi, *et al.* Discovery of a NAPE-PLD Inhibitor That Modulates Emotional Behavior in Mice. *Nat. Chem. Biol.* **2020**, *16* (6), 667–675.

(2) M. P. Baggelaar, F. J. Janssen, A. C. M. van Esbroeck, H. den Dulk, M. Allarà, S. Hoogendoorn, R. McGuire, B. I. Florea, N. Meeuwenoord, H. van den Elst, *et al.* Development of an Activity-Based Probe and In Silico Design Reveal Highly Selective Inhibitors for Diacylglycerol Lipase-α in Brain. *Angew. Chem. Int. Ed.* **2013**, *52* (46), 12081–12085.

(3) Z. Li, P. Hao, L. Li, C. Y. J. Tan, X. Cheng, G. Y. J. Chen, S. K. Sze, H.-M. Shen, and S. Q. Yao Design and Synthesis of Minimalist Terminal Alkyne-Containing Diazirine Photo-Crosslinkers and Their Incorporation into Kinase Inhibitors for Cell- and Tissue-Based Proteome Profiling. *Angew. Chem.* **2013**, *125* (33), 8713–8718.

(4) K. Labun, T. G. Montague, J. A. Gagnon, S. B. Thyme, and E. Valen CHOPCHOP v2: A Web Tool for the next Generation of CRISPR Genome Engineering. *Nucleic Acids Res.* **2016**, *44*, 272–276.

(5) L. Cong, F. A. Ran, D. Cox, S. Lin, R. Barretto, N. Habib, P. D. Hsu, X. Wu, W. Jiang, L. A. Marraffini, *et al.* Multiplex Genome Engineering Using CRISPR/Cas System. *Science* **2013**, *339* (February), 819–824.

(6) F. A. Ran, P. D. Hsu, J. Wright, V. Agarwala, D. A. Scott, and F. Zhang Genome Engineering Using the CRISPR-Cas9 System. *Nat. Protoc.* **2013**, *8* (11), 2281–2308.

(7) A. C. M. Van Esbroeck, V. Kantae, X. Di, T. van der Wel, H. den Dulk, A. F. Stevens, S. Singh, A. T. Bakker, B. I. Florea, N. Stella, *et al.* Identification of α,β-Hydrolase Domain Containing Protein 6 as a Diacylglycerol Lipase in Neuro-2a Cells. *Front. Mol. Neurosci.* **2019**, *12*, 286.

(8) J. V. Peppard, S. Mehdi, Z. Li, and M. S. Duguid ASSAY METHODS FOR IDENTIFYING AGENTS THAT MODIFY THE ACTIVITY OF NAPE-PLD OR Abh4. US20100143937A1, June 10, 2010.

(9) D. Piomelli, P. Magotti, I. Bauer, M. Igarashi, M. Babagoli, R. Marotta, and G. Garau Structure of Human N -Acylphosphatidylethanolamine-Hydrolyzing Phospholipase D: Regulation of Fatty Acid Ethanolamide Biosynthesis by Bile Acids. *Structure* **2015**, *23* (3), 598–604.

(10) A. Chicca, S. Nicolussi, R. Bartholomäus, M. Blunder, A. A. Rey, V. Petrucci, I. del C. Reynoso-Moreno, J. M. Viveros-Paredes, M. D. Gens, B. Lutz, *et al.* Chemical Probes to Potently

and Selectively Inhibit Endocannabinoid Cellular Reuptake. *Proc. Natl. Acad. Sci.* **2017**, *114* (25), E5006–E5015.
